# Transcriptome analysis of cardiac endothelial cells after myocardial infarction reveals temporal changes and long-term deficits

**DOI:** 10.1038/s41598-024-59155-8

**Published:** 2024-05-01

**Authors:** Chitra Basu, Presley L. Cannon, Cassandra P. Awgulewitsch, Cristi L. Galindo, Eric R. Gamazon, Antonis K. Hatzopoulos

**Affiliations:** 1 Division of Cardiovascular Medicine, Department of Medicine, Vanderbilt University Medical Center, Nashville, TN USA; 2 Vascular Medicine Institute, University of Pittsburgh, Pittsburgh, PA USA; 3 Division of Genetic Medicine, Department of Medicine, Vanderbilt University Medical Center, Nashville, TN USA

**Keywords:** Molecular biology, Cardiology, Cardiovascular biology, Myocardial infarction

## Abstract

Endothelial cells (ECs) have essential roles in cardiac tissue repair after myocardial infarction (MI). To establish stage-specific and long-term effects of the ischemic injury on cardiac ECs, we analyzed their transcriptome at landmark time points after MI in mice. We found that early EC response at Day 2 post-MI centered on metabolic changes, acquisition of proinflammatory phenotypes, initiation of the S phase of cell cycle, and activation of stress-response pathways, followed by progression to mitosis (M/G2 phase) and acquisition of proangiogenic and mesenchymal properties during scar formation at Day 7. In contrast, genes involved in vascular physiology and maintenance of vascular tone were suppressed. Importantly, ECs did not return to pre-injury phenotypes after repair has been completed but maintained inflammatory, fibrotic and thrombotic characteristics and lost circadian rhythmicity. We discovered that the highest induced transcript is the mammalian-specific *Sh2d5* gene that promoted migration and invasion of ECs through Rac1 GTPase. Our results revealed a synchronized, temporal activation of disease phenotypes, metabolic pathways, and proliferation in quiescent ECs after MI, indicating that precisely-timed interventions are necessary to optimize cardiac tissue repair and improve outcomes. Furthermore, long-term effects of acute ischemic injury on ECs may contribute to vascular dysfunction and development of heart failure.

## Introduction

Acute myocardial infarction (MI) is one of the leading causes of death. Each year over 800,000 people in the U.S. suffer an MI and most sustain cardiac tissue damage, leading to ventricular remodeling, hypertrophy, dilation, and eventually heart failure (HF)^1^. Percutaneous interventions and thrombolytics aim to restore coronary circulation to the heart after MI^2^, yet there are no current treatments that directly target endogenous repair mechanisms in order to improve cardiac wound healing and prevent HF.

Occlusion of coronary vessels and interruption of oxygen supply result in ischemic death of cardiomyocytes within minutes^3^. Toxic products released from dying cells activate proteases that degrade extracellular matrix (ECM) and induce expression of cytokines and chemokines that recruit neutrophils and monocytes in order to remove necrotic tissue and cellular debris^4^. After the infarct area is cleared, the gap is replaced by granulation tissue that is composed of proliferating cells, mainly myofibroblasts that secrete collagen and other ECM proteins, and endothelial cells (ECs) that build new capillaries to vascularize the newly forming scar tissue^5^.

ECs play diverse roles throughout the cardiac repair process after MI. Besides forming new blood vessels, they induce expression of cell surface cell adhesion proteins that slow down and capture circulating immune cells^4,6^. After the inflammatory phase winds down, a subset of ECs undergoes mesenchymal transition giving rise to myofibroblast-like cells that may contribute to fibrosis^7^.

We have previously shown that manipulation of EC-activated signaling pathways by BMP inhibitors attenuated excessive inflammation after MI, whereas overexpression of canonical Wnt factors enhanced arteriogenesis and tempered fibrosis^6–8^. In both instances, cardiac recovery and functional output improved, suggesting that ECs are an attractive target to enhance cardiac wound healing.

To assess the timing and duration of the phenotypic transformations of quiescent ECs after MI, as well as to discover new regulatory mechanisms of endothelial activation, we isolated and compared the transcriptome of mouse cardiac ECs at Day 0 (D0) without injury to three critical time points, i.e., at 2, 7 and 28 days after MI, using bulk RNA-sequencing. Day 2 (D2) represents early stages of the EC response to ischemic injury; Day 7 (D7) corresponds to the neovascularization and fibrosis stage and, Day 28 (D28) is at the onset of HF after scar tissue has been formed.

We found MI led to acquisition of new transient and long-term characteristics in cardiac ECs. Importantly, ECs did not completely return to their pre-MI state after scar formation has been completed but retained pathological characteristics that may contribute to vascular dysfunction. We discovered that the initial endothelial response involved induction of the mammalian-specific *Src homology 2 (SH2) domain containing 5* (*Sh2d5*) gene. Functional studies in vitro revealed that Sh2d5 promoted EC growth and motility via the Rac1 GTPase pathway.

## Results

### Distinct phases of endothelial activation after MI

**Figure 1:**
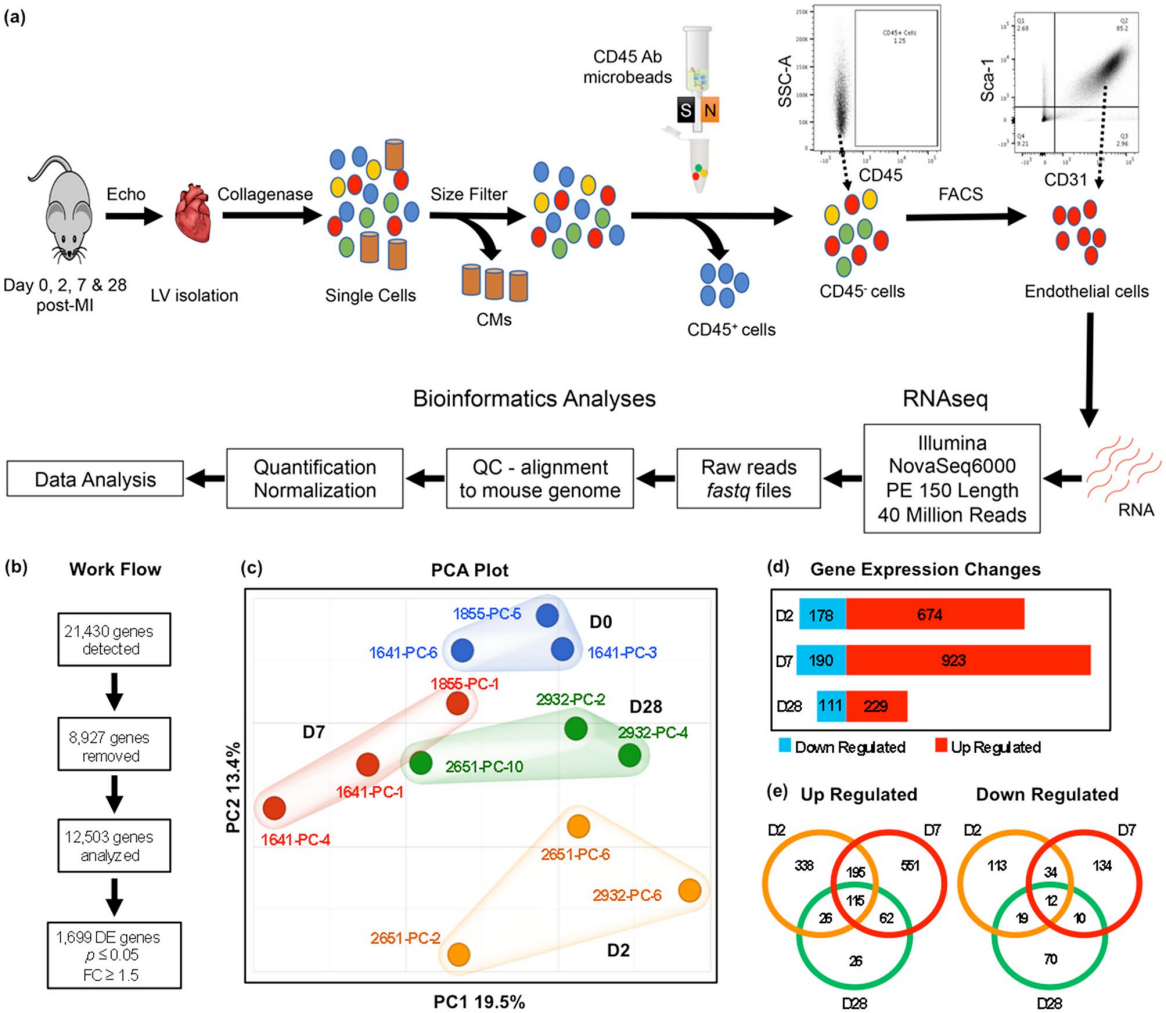
Gene expression changes in endothelial cells after MI. (**a**) Schematic outline of the experimental approach to determine endothelial-specific gene expression changes at distinct stages of tissue repair after MI. CMs: Cardiomyocytes; LV: Left Ventricle; QC: Quality Control. (**b**) Work flow of sequential steps to determine differentially expressed (DE) genes in all examined post-MI time points with at least 1.5-fold change and *p* value ≤ 0.05. (**c**) Principal Component Analysis (PCA) of the 12 bulk RNAseq endothelial datasets indicates clustering according to pre- and post-MI time points. (**d**) Numbers of DE upregulated (red) and downregulated (blue) genes at distinct post-MI time points. (**e**) Venn diagrams depict numbers of stage-specific and shared genes among post-MI time points.

To assess the timing and duration of endothelial phenotypic changes after MI, we used LAD ligation in mice as a model of acute ischemic injury. Whole LVs were isolated at D0 without LAD ligation to obtain baseline expression values, and at D2, D7, and D28 after MI. To isolate pure populations of cardiac ECs, LVs were dissociated to single cells, and cardiomyocytes and CD45^+^ immune cells were removed by size filtration and magnetic cell separation, respectively. The remaining cells, consisting primarily of fibroblasts, pericytes and ECs, were stained with CD31 and Sca1 antibodies. CD31/Sca1 double positive ECs were then isolated as a pure population by FACS and lysed for RNA preparation (Fig. 1a).

To isolate sufficient quantity of high-quality RNA, we pooled together 3–5 hearts per sample and performed 3 independent experiments per time point. Therefore, endothelial expression profiles at each stage of the repair process represent average values from 9 to 15 mice, strengthening the statistical power of experimental results. Echocardiography confirmed infarction and documented increases in diastolic and systolic dimensions and decreases in ejection fraction (EF) and fractional shortening (FS) (Table S1).

RNA samples were submitted to the Vanderbilt VANTAGE core for bulk RNA sequencing and quality controls (QC) (Fig. 1a). Sequence alignment to mouse genome yielded 21,430 transcripts; 8,927 transcripts were removed because all Counts Per Million (CPM) values were 0, or there were less than 3 replicates with values greater than 0 for any sample group, or were pseudogenes, leaving 12,503 expressed genes for further analysis, representing over 90% of the endothelially-expressed genes (Fig. 1b). The CPM values of the 12,503 genes at the examined time points were included in Table S2. Using Partek’s Gene Specific Analysis (GSA) multimodal estimation algorithm to calculate *p*-values and fold changes, 1,699 genes were scored as differentially expressed (DE) at single or multiple time points compared to baseline with *p* ≤ 0.05 and ≥ 1.5-fold up or down changes in expression levels (Fig. 1b; Table S3).

Principal component analysis (PCA) revealed clustering of samples according to post-infarction time point (Fig. 1c). Principal Component 1 (PC1), which accounts for 19.5% of variable gene expression, separated D7 samples along the x-axis, whereas PC2 (13.4% variable), separated D2 samples along the y-axis. PCA also showed that D28 samples clustered in an area between D0, D2 and D7, suggesting that MI led to long-term changes in endothelial phenotypes.

Gene expression changes per stage established that the highest number of genes (1,113) were induced or suppressed at D7, followed by 852 genes at D2 and 340 at D28 (Fig. 1d). The number of upregulated genes was 3.8 × higher than the number of downregulated genes at D2 (674/178), 4.9x (923/190) at D7 and 2.1x (229/111) at D28 (Fig. 1d). Venn diagrams indicated that the majority of gene expression changes at D2 and D7 took place during the corresponding stage, whereas fewer genes were exclusive to D28 (Fig. 1e). A universal core of 127 genes remained induced or suppressed (115/12 genes) throughout all time points post-MI. Overall, our data indicate that the EC response to MI was highly compartmentalized in temporal fashion and pointed to long-term acquisition of MI-related characteristics.

### Quiescent ECs activate proliferative, metabolic, inflammatory, and stress pathways at early post-MI stages (D2)

**Figure 2:**
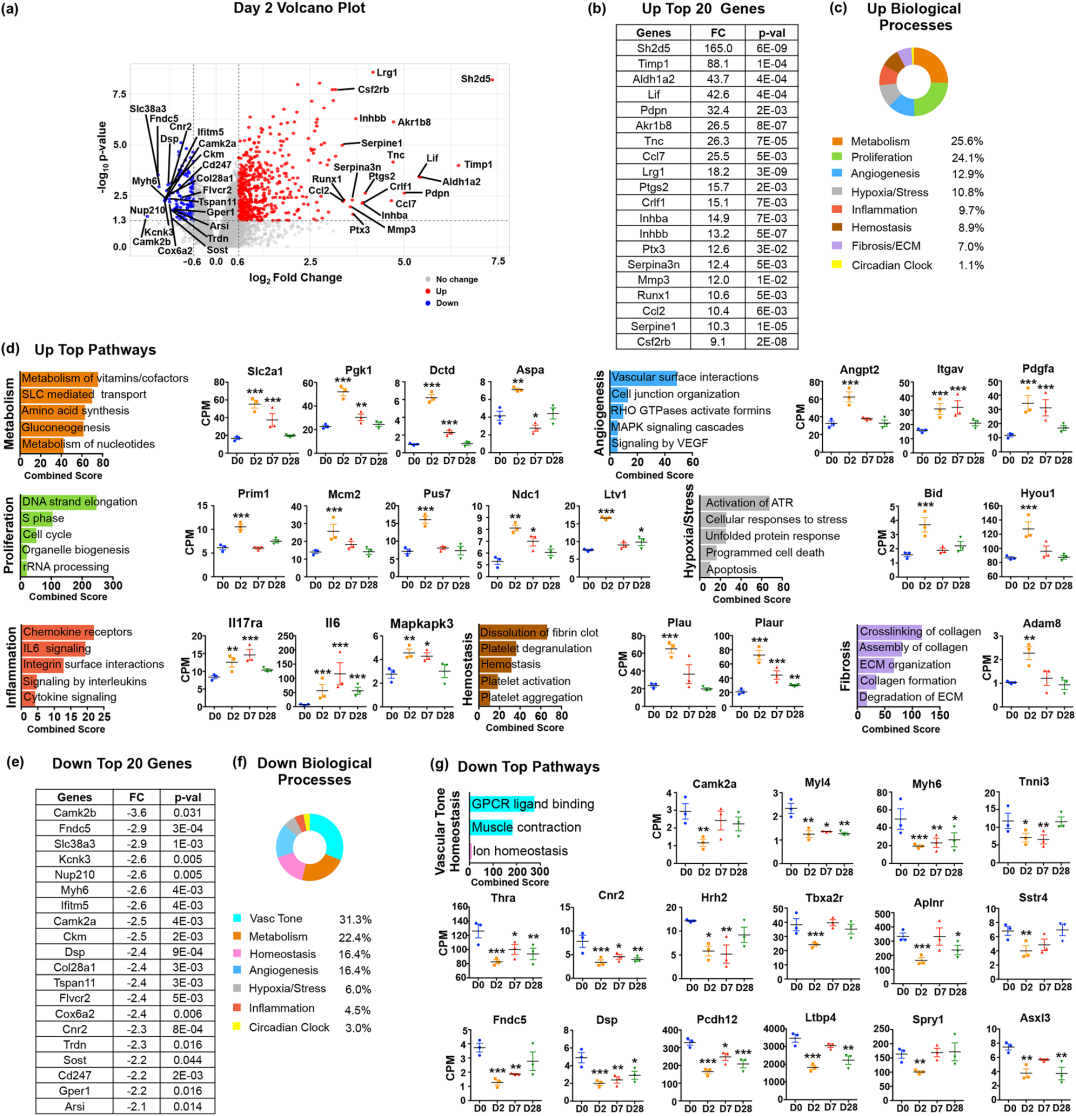
Gene expression changes in endothelial cells at Day 2 after MI. (**a**) Volcano plot of D2 upregulated (in red) and downregulated (in blue) genes with at least 1.5-fold change (FC) and *p* value ≤ 0.05. The 20 upregulated and downregulated genes with the highest induction or suppression FC are marked. (**b**) Table of top 20 upregulated genes at D2 post-MI ordered by FC with the corresponding *p* values. (**c**) Biological Processes associated with D2 upregulated genes ordered by the percentage of genes assigned to each process. (**d**) Graphs of top pathways activated at D2 generated using the Reactome database, ordered by combined score. Pathways were plotted according to their assigned Biological Process. Representative examples of D2 upregulated genes from each biological process are included, n = 3 biological replicates. (**e**) Table of top 20 downregulated genes at D2 ordered by FC with the corresponding *p* values. (**f**) Biological Processes associated with D2 downregulated genes ordered by the percentage of genes in each process. (**g**) Graphs of top pathways decreased at D2 ordered by combined score. Representative examples of D2 downregulated genes are included, n = 3 biological replicates. All gene expression graphs are shown in Counts per Million (CPM) mapped reads, which represent the number of raw reads mapped to the transcript, scaled by the number of sequencing reads, multiplied by a million. Statistical analysis of RNAseq data was performed with Partek’s Gene Specific Analysis (GSA) multimodal estimation. * *p* < 0.05; ** *p* < 0.01; *** *p* < 0.001 comparing expression levels at specific time points to D0 values at baseline without MI.

The Volcano plot in Fig. 2a displays the distribution of DE genes in ECs by fold change and statistical significance at D2 post-MI compared to D0. The top 20 upregulated genes by fold change with corresponding *p* values are shown in Fig. 2b. The highest induced genes encode the pro-inflammatory chemokines Ccl2 (10.4-fold) and Ccl7 (25.5-fold), which stimulate ECs and act as chemoattractants for monocytes and neutrophils^9^; the common subunit beta of the Colony stimulating factor 2 receptor (Csf2rb, 9.1-fold); the pro-inflammatory enzyme Prostaglandin-Endoperoxide Synthase 2, also known as Cyclooxygenase 2 (Ptgs2 or Cox-2, 15.7-fold); and Pentraxin 3 (Ptx3, 12.6-fold) that is important for clearance of apoptotic cells^10^. The top group also included the Leukemia Inhibitor Factor (Lif; 42.6-fold), which contributes to the inflammatory stimulation of ECs through Stat3 activation and also protects myocardium and ECs from acute oxidative stress^11^. It further contained genes for proteins involved in ECM degradation and remodeling such as Matrix metalloproteinase 3 (Mmp3, 12-fold), Tissue inhibitor of metalloproteases 1 (Timp1, 88-fold) and Tenascin-c (Tnc, 26.3-fold); pro-angiogenic factors Inhibin A and B (Inhba, 14.9-fold; Inhbb, 13.2-fold), Leucine Rich Alpha-2-Glycoprotein 1 (Lrg1, 18.2-fold), and the transcription factor Runx1 (10.6-fold); and, enzymes involved in metabolism and regulation of oxidative stress such as the Aldehyde dehydrogenase 1 family, member A2 (Aldh1a2; 43.7-fold) and Aldose reductase-related protein 2 (Akr1b8; 26.5-fold) (Table 1).

**Table 1:** Day 2 post-MI gene expression changes and the corresponding biological processes and cellular functions.

Day 2 post-MI upregulated genes and biological processes in endothelial cells					
Metabolism	Proliferation/Growth	Angiogenesis/Motility	Inflammation	ECM/Fibrosis	Hypoxia/Stress
*Glucose metabolism*	*Cell Cycle/S-phase/DNA replication*	*Angiogenesis regulators*	*Chemokines/Cytokines*	*Degradation/Remodeling*	*Oxidative stress*
**Eno1**, **Gapdh**, Pgk1*, **Pgam1**, **Pgm3**, **Pkm**, **Pygl**, **Tpi1**	**Brca2**, Ccne2, **Cdc7**, Cdc45, Mcm2*, **Mcm3**, **Mcm5**, Myc**, Plk4, Pola1**, **Pold2**, Pole, Pole4 Prim1*, **Prim2**, **Rfc4**	Angpt2*, Apln, **Hbegf**, **Inhba**, Inhbb, Lrg1**, Pdgfa*, Pgf, Runx1, Vegfd	**Ccl2**, Ccl7, Cxcl1, Cxcl2, Cxcl10, Il6*, Il33, Lif, **Mif**	Adam8*, Adam12, Adamts4, **Adamts9**, Loxl2, Loxl3, Loxl4, Mmp3, Mmp14, Timp1	Akr1b8, Aldh1a2, **Edn1**
*Glucose transporters*	*RNA synthesis*	*Cell motility/Migration*	*Receptors/Signaling*	*ECM proteins*	*Hypoxia*
Slc2a1* (Glut1), **Scl2a3** (Glut3)	Polr2h, **Polr3d**, **Polr3g**, **Polr3k**	**Dock5**, Dock8, Itga2, **Itga2b**, Itga3, Itgb3, **Itgb3bp**, Itgav*, Sema3f, Slit2, Tubb6	Csf2rb, Csf2rb2, Il4ra, Il17ra*, Lrg1**, Mapkapk3*, Tnfrsf12a, Tnfrsf23, **Tlr4**	Col3a1, Col5a3, Col6a3, Col27a1, Lamc2, Thbs1, Tnc	Hif1a, Hyou1*
*Amino acid transporters*	*RNA processing and transport*		*Cell adhesion/Recruitment*	*Profibrotic factors*	*Stress/Apoptosis*
Slc1a4, Slc1a5, **Slc3a2**, Slc7a5, Slc16a3, Slc38a1, Slc43a1	**Fbl, Mettl1**, Pus7*, Ndc1*, **Nupl2**, **Nup37**, **Nup43**, **Nup205**		Plvap**, Sele**, Selp**, Vcam1**	Ctgf, **Edn1**	Bid*, Bnip3, Casp3, Casp4, **Gdf15**, **Hspa5**, **Hspa9**, **Hspa13**, **Ripk3**, **Psmc4**, **Psmc6**, **Psmd14**

				**Hemostasis/Thrombosis**	**Circadian Clock**
*Membrane transporters*	*Ribosome biogenesis/Translation*		*Proinflammatory enzymes*	*Coagulation/Thrombosis*	*Circadian pathway*
**Slc12a8**, Slc19a2, Slc20a1, **Slc25a13**, Slc39a6, Slco2a1	Brix1, Eef1e1, Eif1a, Eif2b3, Eif5a, Ltv1*, **Tsr1**, **Wdr12**		Pla2g4a, Ptgis***, Ptgs2 (Cox-2), Ptx3	Anxa2, **Pf4**, Plau*, Plaur*, Pdpn, Sele**, Selp**, Serpine1*** (PAI-1)	Bhlhe41***, Dbp***, Hlf***, Hif1α Nr1d1*** (Rev-erbα), Nr1d2*** (Rev-erbβ), Per3***, Tef***
*Nucleotide biosynthesis*	*Mitochondrial ribosomal proteins*		*Immune response*		
Dctd*, **Dhfr**, **Mthfd1**, Mthfd2, Mthfd1l, Tyms	**Mrpl50**, **Mprl52**, **Mrpl54**, **Mrps10**, **Mrps22**, **Mrps25**, **Mrps27**, Mrps36		Anxa1		
*Amino acid biosynthesis*	*Cell growth*		*Prostaglandin biosynthesis*		
Aldh18a1, **Asns**, Aspa*, **Got1**, **Shmt2**	**Ngf, Src**		Ptgis***, Ptgs2 (Cox-2)		

Day 2 post-MI downregulated genes and biological processes in endothelial cells					
Vascular tone	Vascular homeostasis	Angiogenesis/Motility	Signaling regulation	Metabolism	
*Ca*^***2****+*^*signaling/Handling/Contraction*	*GPCR receptors*	*Cell adhesion/Vessel stability*	*Negative regulators*	*Membrane transporters*	
Atp1a2, Atp1b2, Camk2a*, Camk2b, **Kcnb1**, **Kcnj15**, Kcnk3**, Trdn**	**Adora2a***,* Aplnr*, Cnr2*, Gper1, Hrh2*, Sstr4*, Tbxa2r*	Cdh23, Dsp*, Fndc5*, **Tspan11**, Pcdh12*	Asxl3*, **Ctnnbip1**, **Hes1**, **Hopx**, Ltbp4*, Plekhh2, Sost, Spry1*	Aqp1, Aqp7, **Slco2b1**, Slc38a3	

				**Hypoxia/Stress**	
*Filament proteins/Contraction*	*Nuclear hormone receptors*	*Migration/Angiogenesis*		*Stress/Apoptosis*	
**Actn2**, **Mybpc3**, Myh6*, Myl2, Myl4*, **Myo10**, Myom2, Myoz2, Ttn, Tnni3*	Thra*	Igf2, Kdr (Vegfr2), Robo2, **Sema6c**		Arntl (Bmal1),** Pycard**	

				**Circadian Clock**	
				*Circadian pathway*	
				Arntl (Bmal1)	

*Marks genes whose post-MI expression patterns are depicted in Fig. 2.

**Figure 3:**
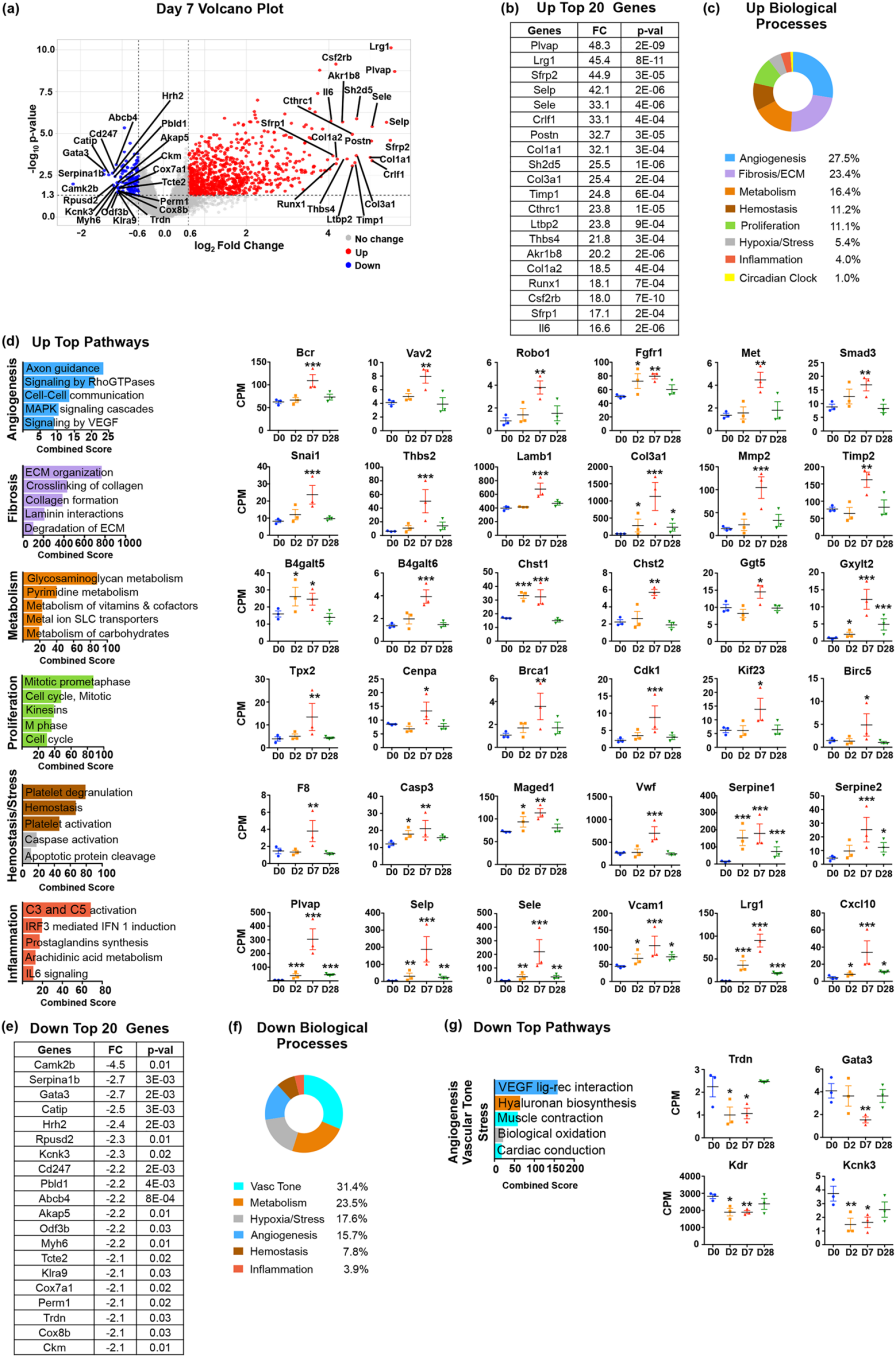
Gene expression changes in endothelial cells at Day 7 after MI. (**a**) Volcano plot of D7 upregulated (in red) and downregulated (in blue) genes with at least 1.5 FC and *p* value ≤ 0.05. The 20 upregulated and downregulated genes with the highest induction or suppression FC are marked. (**b**) Table of top 20 upregulated genes at D7 ordered by FC with the corresponding *p* values. (**c**) Biological Processes associated with D7 upregulated genes ordered by the percentage of genes assigned to each process. (**d**) Graphs of top pathways activated at D7 generated using the Reactome database, ordered by combined score. Pathways were plotted according to their assigned Biological Process. Representative examples of D7 upregulated genes from each biological process are included, n = 3 biological replicates. (**e**) Table of top 20 downregulated genes at D7 ordered by FC with the corresponding *p* values. (**f)** Biological Processes associated with D7 downregulated genes ordered by the percentage of genes in each process. (**g**) Graphs of top pathways decreased at D7 ordered by combined score. Representative examples of D7 downregulated genes are included, n = 3 biological replicates. CPM: Counts per Million mapped reads. Statistical analysis of RNAseq data was performed with Partek’s Gene Specific Analysis (GSA) multimodal estimation. * *p* < 0.05; ** *p* < 0.01; *** *p* < 0.001 comparing expression levels at specific time points to D0 values.

**Marks genes whose post-MI expression patterns are depicted in Fig. 3.

**Figure 4:**
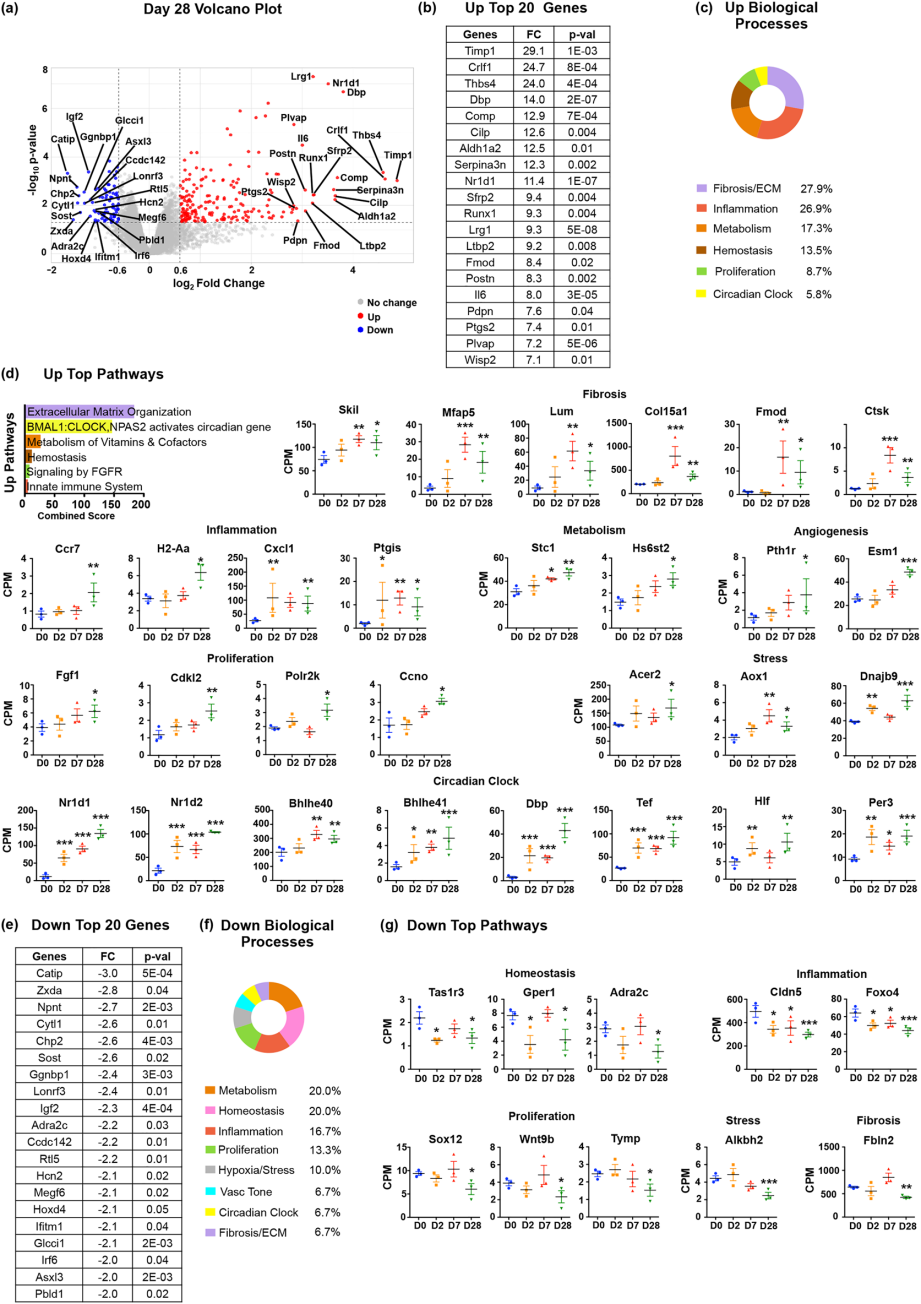
Gene expression changes in endothelial cells at Day 28 after MI. (**a**) Volcano plot of D28 upregulated (in red) and downregulated in (blue) genes with at least 1.5-FC and *p* value ≤ 0.05. The 20 upregulated and downregulated genes with the highest induction or suppression fold changes are marked. (**b**) Table of top 20 upregulated genes at D28 ordered by FC with the corresponding *p* values. (**c**) Biological Processes associated with D28 upregulated genes ordered by the percentage of genes assigned to each process. (**d**) Graph of top pathways activated at D28 generated using the Reactome database, ordered by combined score. Pathways were plotted according to their assigned Biological Process. Representative examples of D28 upregulated genes from each biological process are included, n = 3 biological replicates. (**e**) Table of top 20 downregulated genes at D28 ordered by FC with the corresponding *p* values. (**f**) Biological Processes associated with D28 downregulated genes ordered by the percentage of genes in each process. (**g**) Representative examples of D28 downregulated genes are included, n = 3 biological replicates. CPM: Counts per Million mapped reads. Statistical analysis of RNAseq data was performed with Partek’s Gene Specific Analysis (GSA) multimodal estimation. * *p* < 0.05; ** *p* < 0.01; *** *p* < 0.001 expression levels at specific time points to D0 values.

***Marks genes whose post-MI expression patterns are depicted in Fig. 4.

Genes in Bold are primarily induced or suppressed at D2.

Underlined genes represent upregulated genes whose post-MI expression levels did not return to baseline.

The 674 upregulated genes at D2 are depicted in Table S4, which also includes the subset of 338 genes that were primarily induced at D2. Using the Reactome online database (https://maayanlab.cloud/Enrichr/#), we found that upregulated genes were associated with 245 pathways with at least two representative gene members and *p* value ≤ 0.05. The majority of the pathways could be further assembled in eight biological processes related to metabolism, cell growth and proliferation, angiogenesis and cell motility, hypoxia and cellular stress, inflammation, hemostasis and thrombosis, ECM remodeling, and circadian clock (Table S5).

Based on the number of unique genes in each group relative to the total number of assigned genes at D2, we calculated the contribution of gene expression changes to various biological processes in D2 ECs (Fig. 2c). Overall, approximately 60% of D2 induced genes took part in metabolism, cell growth, motility and angiogenesis, whereas the remaining ~ 40% participated in stress responses to tissue damage and hypoxia and early repair processes triggered by the injury, i.e., inflammation, ECM degradation, and thrombosis.

### Metabolic changes, entry to the S-phase of the cell cycle, and initial activation of pro-angiogenic processes dominate early EC responses to MI

The top upregulated category at D2 consisted of genes linked to metabolic pathways (Fig. 2c). They primarily encoded enzymes involved in glucose metabolism and biosynthesis of nucleotides needed for DNA replication, amino acids for protein synthesis, as well as prostanoids that are critical for regulation of inflammation, thrombosis and vascular tone^12^. There was also a sizable number of membrane transporters for glucose and amino acids (Fig. 2d; Table 1).

The second largest category contained genes that take part in different aspects of cell growth (Fig. 2d; Table 1). It is notable that the majority of genes linked to proliferation were associated with the early stages of the cell cycle or the S-phase/G1 transition and the initiation of DNA replication, RNA synthesis, RNA processing and export to the cytoplasm, cytoplasmic and mitochondria ribosomal biogenesis and assembly, and RNA translation.

Genes encoding pro-angiogenic factors and stimulators of endothelial motility also featured prominently (Fig. 2d; Table 1), including the Tie-2 ligand Angiopoietin 2 (Angpt2) that disrupts vessel stability, a first step in angiogenesis^13^. Taking together, these results highlight the transformation of quiescent ECs to rapidly growing cells.

### Activation of stress responses and repair mechanisms in early post-MI ECs

Next to changes related to EC growth, early activation phenotypes centered on stress and cell death related genes, such as the Hypoxia-induced factor 1α (Hif1α) and Caspase 3 (Casp3)^14,15^. Early activation also involved acquisition of proinflammatory characteristics through induction of genes encoding surface cell adhesion molecules to slow down and capture circulating immune cells, chemokines and cytokines for recruitment and expansion of pro-inflammatory cells, as well as signaling components of proinflammatory pathways. In addition, this group contained genes involved in ECM degradation and remodeling; and, in platelet reactivity and coagulation, that may contribute to prothrombotic phenotypes in post-MI ECs (Fig. 2d; Table 1).

To recognize spatial induction patterns of pro-inflammatory cell surface cell adhesion proteins such as Sele in endothelial cells after MI, we co-stained cardiac tissue sections with antibodies recognizing Sele protein and the typical endothelial marker CD3l. Immunofluorescence imaging showed that at D0 Sele was expressed in universal fashion in ECs throughout the heart, similarly to CD31 (Fig. S1a). At D2 post-MI, Sele protein was induced in most ECs, both in and around the infarct and peri-infarct areas, as well as at distal sites. However, relative induction levels relative to CD31 were higher in and around the infarct area than at distal sites (Fig. S1b, c).

### MI disrupts homeostatic functions of ECs

In comparison to the considerable induction levels in upregulated genes, expression changes in D2 downregulated genes were modest and concerned genes with relatively uncharacterized roles in ECs (Table S6). The list of the top 20 downregulated genes by fold change contained transcripts that encode filament proteins and Ca^2+^ signaling components, e.g., Calcium/Calmodulin dependent kinases Camk2b (− 3.6-fold) and Camk2a (− 2.5 fold); Myosin heavy chain 6 (Myh6, − 2.6 fold); Potassium two pore domain channel subfamily K member 3 (Kcnk3, − 2.6 fold); and Triadin (Trdn, − 2.3-fold), suggesting that MI disrupts EC contraction and vascular tone^16^ (Fig. 2e–g; Table 1).

Genes suppressed at D2 also included nuclear receptors and membrane G Protein-Coupled receptors (GPCR) for various hormones, indicating that regulation of endothelial homeostatic functions, such as NO production, vasodilation and permeability was compromised after MI (Table 1). Expression of a subset of hormone receptors and other down regulated genes did not return to pre-MI levels later-on, suggesting that vascular homeostatic functions were only partially restored in cardiac ECs after MI (Fig. 2g).

There was also significant suppression of the *Fibronectin type III domain-containing protein 5* gene (*Fndc5*; − 2.9-fold) (Fig. 2e, g). *Fndc5* encodes the precursor of the newly discovered hormone Irisin, an exercise-induced stimulator of muscle metabolism. Irisin also promotes formation of EC junctions, therefore downregulation of Irisin likely causes breakdown of cell adhesion and vessel stability, increasing EC mobility^17^. This is further supported by the fact that other genes encoding EC adhesion proteins were downregulated, including Desmoplakin (Dsp; − 2.4-fold), Tetraspanin 11 (Tspan11; − 2.4-fold) and the cell-adhesion VE-cadherin 12 protein (Pcdh12; − 2-fold) (Fig. 2g; Table 1).

The 178 downregulated genes at D2 are shown in Table S6, which also includes the subset of 113 genes that are primarily suppressed at D2. To calculate the relative contribution of the endothelial gene expression changes to the compromised biological processes, we used the Reactome pathway database (Table S7). Overall, about half of downregulated genes played roles in regulation and maintenance of vascular tone and vascular homeostasis, and the remaining in metabolism, angiogenesis and cell motility, hypoxic and cellular stress, inflammation and circadian clock (Fig. 2f).

Importantly, genes suppressed at D2 also encoded negative regulators of key signaling pathways and cellular processes. Among them were Sclerostin (Sost), an inhibitor of Wnt signaling, a pathway involved in multiple processes of wound healing after MI^7,8^; Sprouty RTK signaling antagonist 1 (Spry1) a repressor of Fibroblast Growth Factor (FGF) signaling, which promotes EC proliferation and angiogenesis^18^; Hes family bHLH transcription factor 1 (Hes1), a Notch signaling target and repressor of factors required for EC growth and angiogenesis^19^; HOP homeobox (Hop) that inhibits the transcription factor Serum Response Factor (SRF), which promotes cell growth and migration^20^; Latent transforming growth factor beta binding protein 4 (Ltbp4) that prevents activation of TGFβ signaling, a key pathway in fibrosis and resolution of inflammation^21^; ASXL transcriptional regulator 3 (Asxl3), a transcriptional inhibitor that negatively regulate lipogenesis^22^; and, Pleckstrin homology domain-containing factor 2 (Plekhh2), which blocks actin filament depolymerization, thereby stabilizing filaments and preventing proliferation and migration^23^ (Fig. 2g; Table 1). Together, the data suggest that suppression of negative regulators of major signaling pathways may be an important component of endothelial activation after MI.

### Programs of cardiac repair are activated in ECs during granulation tissue formation (D7)

The overall distribution of deregulated genes at D7 compared to D0 is presented by Volcano plot (Fig. 3a). The 923 upregulated genes at D7 are displayed in Table S8, which also includes the subset of 551 genes that are primarily induced at D7. The top 20 D7 upregulated genes by fold change are shown in Fig. 3b. The highest induced gene during the neovascularization stage was the *Plasmalemma vesicle-associated protein* gene (*Plvap*; 48-fold), which encodes an EC-specific structural protein of caveolae fenestrae and trans-endothelial channels that controls vessel permeability^24^. Based on the vascular deficits observed in embryos lacking Plvap, it is likely that increased Plvap expression promotes the integrity of newly forming blood vessels. The pro-inflammatory phenotypes of ECs persisted as manifested by continued induction of genes encoding Sele, Selp and Interleukin 6 (Il6). Moreover, and in contrast to D2, the top 20-induced genes encoded proteins involved in ECM synthesis and deposition, including several collagens (Col1a1, 32-fold; Col3a1, 25-fold; Col1a2, 18.5-fold), Periostin (Postn, 38-fold), and Thrombospondin 4 (Thbs4, 22-fold), as well as Secreted frizzled related proteins 1 & 2 (Sfrp1 17-fold; Sfrp2 45-fold), two Wnt signaling antagonists known to promote fibrosis^25^.

Reactome database analysis of D7 upregulated genes identified 201 pathways with significant *p* value ≤ 0.05 that were further classified in biological processes (Table S9). The relative contribution of genes with expression changes in each biological process revealed that approximately half of D7 activated genes were involved in angiogenesis and ECM production, the other half in metabolism, hemostasis and thrombosis, proliferation, hypoxia and cellular stress, inflammation and circadian clock (Fig. 3c).

### ECs enter mitosis and acquire angiogenic phenotypes during scar tissue formation

The complexity of pathways necessary for neovascularization of the newly forming scar tissue greatly increased during this stage (Fig. 3d; Table S9). Prominent among signaling components were upstream regulators and downstream targets of Rac and Rho GTPase signaling (Table 2). Rho-family proteins are key regulators of angiogenesis, modulating a diversity of cellular processes, including vascular permeability, actin remodeling, invasion, migration, ECM remodeling, proliferation, morphogenesis, and survival^26^. There were also a number of induced components in Robo-Slit and Semaphorin-Plexin-Neuropilin pathways that act upstream of Rho GTPases and are critical for cell migration, angiogenesis, and blood vessel assembly^27,28^. Additional active pathways included Wnt signaling, which is important for endothelial growth and vessel formation, MAPK signaling that is critical for cell proliferation, migration and angiogenesis, Hedgehog signaling, which plays a role in vascular integrity, Insulin signaling that promotes vascular formation, and Tgfβ family signaling that is known to modulate vascular remodeling in response to injury and to be involved in inflammation and fibrosis^29^ (Table 2). The expression of the majority of genes linked to angiogenesis was restricted to D7.

**Table 2:** Day 7 post-MI gene expression changes and the corresponding biological processes and cellular functions.

Day 7 post-MI upregulated genes and biological processes in endothelial cells					
Angiogenesis/Motility	ECM/Fibrosis	Proliferation/Growth	Metabolism	Inflammation	Hypoxia/Stress
*Migration/Tube formation*	*ECM proteins*	*Cell Cycle/Mitosis*	*Protein modification enzymes*	*Chemokines/Cytokines*	*Oxidative stress*
**Dock7**, Dock8, **Grik5**, Itga2, Itga3, **Itga5**, **Itga11**, Itgav*, Itgb3, **Itgb4**, Itgbl1, **Plxna3**, **Plxnb2**, Robo1**, Sema3c, **Sema3d**, Sema3f., **Sema6b**, Slit2, **Slit3,** Tubb6	**Col1a1**, **Col1a2**, Col3a1**, **Col4a1**, **Col4a2**, **Col4a5**, **Col5a1**, **Col5a2**, Col5a3, **Col6a1**, **Col6a2**, Col6a3, **Col12a1**, **Col14a1**, Col15a1, **Col16a1**, **Col18a1**, Col27a1, Fbln1, **Fbln5**, **Fn1**, **Lama2**, Lamb1**, **Lamc1**, Lamc2, **Mfap2**, Mfap4, Mfap5***, Postn, Thbs1, Thbs2**, **Thbs3**, Thbs4, Tnc, Vcan	Birc5**, Brca1**, **Bub1**, **Bub1b**, **Ccna2**, **Ccnb2**, Ccne2, **Cdc20**, Cdc45, **Cdca8**, Cdk1**, **Espl1**, **Hmmr**, **Kif11**, **Kif18b**, **Kif20a**, Kif20b, Kif22, Kif23**, **Kifc1, Plk1**	B4galt5**, B4galt6**, **Chpf**, Chst1**, Chst2**, Gale, **Glb1**, Ggt5**, Gxylt2**, **Has2**, **Hs3st1**	Ccl7, Cxcl1, Cxcl2, Cxcl10, **Cxcl14**, Il6*, Il33, Lif	Akr1b8, Aldh1a1, Aldh1a2
*Angiogenesis*	*ECM processing/collagen-crosslinking enzymes*	*Microtubules/Chromosome assembly & segregation*	*Membrane transporters*	*Receptors/Signaling*	*Hypoxia*
**Lepr**, Lrg1**, **Mdk**, **Sdc2**, Sdc4	Adam12**, Adamts2,** Adamts4**, Adamts8, Bmp1,** Ctsk***, **Lox**, Loxl1, Loxl2, Loxl3, Loxl4, Mmp2**, Mmp14, **Mmp19**, **Mmp23**, Timp1, Timp2**	**Ncapd2**, **Ncapg**, **Ncapg2**, **Ncaph**, **Nek2**, Tpx2**, **Top2a**	Slc1a4, Slc1a5, **Slc12a2**, Slc16a3, Slc19a2, Slc2a1*, **Slc2a13**, Slc20a1, **Slc22A4**, **Slc29a1**, **Slc31a1**, Slc38a1, Slc38a2, Slc39a6, **Slc39a8**, **Slc40a1**, Slc41a2, Slc43a1, **Slc7a2**, Slc7a5, **Slc7a7**, Slco2a1	**Ackr2**, Csf2rb, Csf2rb2, IL4ra, Il17ra*, Lrg1**, Mapkapk3*, Tnfrsf12a, Tnfrsf23, **Il13ra1**	Hif1α
*Rac and Rho GTPase signaling*	*Collagen fibril assembly*	*Kinetochore assembly/Sister chromatid separation*	*Nucleotide biosynthesis*	*Cell adhesion & Recruitment*	*Apoptosis*
**Arhgap11a**, **Arhgap24**, **Arhgap44**, Bcr**, **Chn1**, **Cit**, **Diaph3**, **Ect2**, **Fgd1**, **Iqgap3**, **Pak4**, **Pkn3**, **Racgap1**, **Rhou**, **Rnd1**, Vav2**	**Bgn**, **Dcn**, Fmod***, Lum***, **Ogn**	**Aurkb**, Cenpa**, **Cenpe**, **Cenpf**, Cenpq**,** Cenpt, **Incenp**, **Nuf2, Spc24**	Dctd*, Mthfd2, Mthfd1l, Tyms	**Icam1**, Plvap**, Sele**, Selp**, Vcam1**	Casp3
*Wnt signaling*	*Profibrotic factors*		*Amino acid biosynthesis*	*Prostaglandin biosynthesis*	
**Dact1**, **Dact3**, Dkk3, **Frat2**, **Fzd2**, **Gpc3**, **Gpc6**, **Ror1**, **Ror2**, Sfrp1, Sfrp2, **Sox4**, **Sox9**, **Wls (Evi)**	Ctgf, Ltbp2, **Ltbp3,** Sfrp1, Sfrp2, Skil***, Smad3**, **TGFβ3**		Aldh18a1	**Ltc4s**, Pla2g4a, Ptgis***, **Ptgr1**, Ptgs2 (Cox-2), Ptx3	

				**Hemostasis/Thrombosis**	**Circadian Clock**
*TK Receptors/MAPK signaling*	*EndMT*			*Coagulation/Thrombosis*	*Circadian pathway*
**Egfr**, Fgfr1**, Met*, **Rasa3**, **Rasa4**, **Ret**	**Cdh2,** Glipr2, Snai1**, Vim			Anxa2, Plaur*, Pdpn, Sele**, Selp**, Serpine1*** (PAI-1), Serpine2***, **vWF**	Bhlhe40***, Bhlhe41***, Dbp***, Hif1α, Nr1d1***, Nr1d2*** , Per3***, **Ror1**, **Ror2**, Tef***
*Hedgehog signaling*					
**Arrb1**, **Cdon**, **Dhh**, **Evc2**, **Gas1**, **Gli3**, Ift122					
*Insulin signaling*					
**Igf1**, **Igfbp4**, **Igfbp5**, **Igfbp6**, **Igfbp7**					
*Tgfβ signaling*					
**Fstl1**, Skil***, **Smad3, TGFβ3**					
*BMP signaling*					
Bmp2, **Bmp2k**, Bmp4, **Bmper**, Bmpr1a					

Day 7 post-MI downregulated genes and biological processes in endothelial cells					
Vascular tone	Vascular homeostasis	Angiogenesis/Motility	Metabolism	Hypoxia/Stress	Circadian clock
*Ca*^***2****+*^*signaling/Handling/Contraction*	*GPCR receptors*	*Cell adhesion/Vessel stability*	*Membrane transporters*	*Oxidative stress*	*Circadian pathway*
Camk2b, Kcnk3**, **Pln**, Trdn**	Cnr2*, Hrh2*	Cdh23, Dsp*, Fndc5*	Aqp7, **Slc1a1,** Slc16a11	**Chac1, Cyp2u1, Cyp4b1, Cyp2d22, Fmo2**, **Fmo5**, **Gstm1**, **Gstt1**, **Gstt2**	Arntl (Bmal1)
*Filaments/Contraction*	*Nuclear hormone receptors*	*Migration/Angiogenesis*	*ABC transporters*	*Stress/Apoptosis*	
Myh6*, Myl2, **Myl3**, Myl4*, Mylk3, Myom2, Myoz2, Ttn, Tnni3	Thra*	Bmp6, Gata3**, Kdr** (Vegfr2), Robo2, **Tgfb2**, **Vegfc**	**Abcb1a**, **Abcb1b**, **Abcb4**, **Abcb6**, Abcb9	Arntl (Bmal1)	
		*Rac and Rho GTPase signaling*	*N-glycan biosynthesis*	*DNA damage repair*	
		**Arap2, Arhgap18, Rasgrf2**	**Dpm1, St8sia2, St8sia4**	**Chek2**, **Hist1h2be**, **Hist2h2be**, **Rnf8**	
			*Arachidonic acid metabolism*		
			**Alox12, Cyp2u1, Cyp4b1, Cyp2d22**		

*Marks genes whose post-MI expression patterns are depicted in Fig. 2.

**Marks genes whose post-MI expression patterns are depicted in Fig. 3.

***Marks genes whose post-MI expression patterns are depicted in Fig. 4.

Genes in Bold are primarily induced or suppressed at D7.

Underlined genes represent genes whose post-MI expression levels did not return to baseline.

Transcripts induced at D7 also included a large number of genes linked to the cell cycle. However, unlike genes induced at D2 that were involved in DNA synthesis and replication during the S phase of the cycle, D7 induced genes take part in mitosis and cell division during the G2/M phase transition (Fig. 3d; Tables S8, S9). The D7 group included genes encoding proteins involved in microtubule formation, chromosome assembly, condensation and segregation, kinetochore assembly, sister chromatid cohesion and separation, construction of the mitotic spindle to ensure chromosome congression and segregation, maintenance of mitotic spindle integrity, and cleavage furrow formation.

There were also significant changes in metabolic processes. However, the upregulation of enzymes involved in glycolytic pathways and glucose transporters at D2 had retreated, as were enzymes involved in nucleotide and amino acid biosynthesis and ribosome biogenesis. In contrast, D7 metabolic changes involved upregulation of enzymes that take part in posttranslational modification of proteins, indicative of robust synthesis and maturation of membrane and extracellular proteins that are engaged in the construction of new vascular beds and scar tissue (Fig. 3d; Table 2).

### ECs upregulate ECM production after MI

Consistent with our lineage tracing studies that showed Endothelial-to Mesenchymal-Transition (EndMT) takes place during scar formation after MI^7^, we found induction of genes with prominent roles in EndMT, such as *Snail* 1 (*Snai1*) and *Vimentin* (*Vim*) (Fig. 3d; Table 2; Table S8). Moreover, a large number of genes encoding basement membrane and ECM proteins were induced in ECs, including proteins involved in collagen synthesis, fibril assembly and cross-linking, metalloproteases and metalloprotease inhibitors, laminins, and thrombospondins (Fig. 3d; Table 2). Together, upregulation of this extensive repertoire of ECM components and ECM processing enzymes suggests that, besides fibroblasts and macrophages^30,31^, ECs also contribute to ECM deposition after MI.

### Proinflammatory endothelial phenotypes persist during scar tissue formation

As evident from the top 20 list of upregulated genes at D7 (Fig. 3b), the proinflammatory phenotypes of ECs persist at D7 with increased levels of genes encoding immune cell binding proteins (Vcam1, Icam1) and chemokines (Fig. 3d; Table 2). These results suggest that recruitment of circulating immune cells by endothelial cells continued after most debris have been cleared. Studies in many tissues after injury have established that immune cells, e.g., neutrophils, mast cells, macrophages and lymphocytes play important roles in fibrosis^5^. It is likely that delivery of immune cells during late repair stages is critical for scar formation, highlighting an indirect contribution of ECs to cardiac fibrosis. Moreover, a feature of endothelial phenotypes at D7 was the induction of a group of metabolic enzymes that are required for prostaglandin biosynthesis. Prostaglandins have both pro- and anti-inflammatory effects, act as vasodilators to improve blood flow, and are involved in thrombosis^32^. Through their combined actions, prostaglandins are important modulators of wound healing.

It is noteworthy that although expression of proinflammatory molecules persisted at D7, there were also signs that proinflammatory processes gradually subsided as suggested by the upregulation of genes for proteins involved in the resolution of inflammation such as the Atypical Chemokine Receptor 2 (Ackr2), which is scavenging chemokines, the Suppressor of Cytokine Signaling 3 (Socs3), the receptors Il4ra and Il13ra1 that respond to anti-inflammatory cytokines Interleukin 4 and 13 (Il4, Il13), and the anti-inflammatory (and proangiogenic) BMP Endothelial cell Precursor-derived regulator (Bmper) (Table 2)^33,34^.

### Endothelial homeostatic functions remain compromised during scar formation

Gene and pathway analyses showed that the downregulation of genes involved in contractility and vascular tone observed at D2 extended to D7 (Fig. 3e–g; Table 2; Tables S10, S11). The expression of hormone receptors that regulate homeostatic functions was also suppressed as at D2, with the exception of Thromboxane a2 receptor (Tbxa2r). At D7, there was also a decrease in the levels of genes encoding proteins involved in angiogenesis such as Vegfr2 and Vascular endothelial growth factor C (Vegfc), as well as Gata3, which is essential for mediating Tie2 expression (Fig. 3g; Table 2)^35^. These results suggest that signaling pathways involved in the induction of angiogenesis began to wind down, whereas genes taking part in assembly and maturation of blood vessels took over.

Together, our data show that DE genes during scar formation at D7 were mostly divided between genes that support blood vessel formation and ECM deposition. The data also corroborate lineage tracing studies, indicating a prominent mesenchymal transformation of ECs after MI^7^. Finally, it appears that during scar tissue formation, ECs maintain proinflammatory phenotypes, whereas at the same time most genes related to stress and hypoxia have retreated.

### Long-term effects of myocardial infarction on ECs

PCA indicated that D28 ECs did not fully return to baseline suggesting that ECs retained long-term MI-caused phenotypic characteristics, even after scar formation has been completed (Fig. 1). The distribution of D28 DE genes is highlighted in the corresponding Volcano plot (Fig. 4a). The top 20 up-regulated genes are shown in Fig. 4b. The genes that were expressed higher than baseline at this late post-MI stage are listed in Table S12. It includes ECM proteins and receptors, ECM-processing enzymes and profibrotic regulators (Table 3). It also includes genes indicative of a sustained proinflammatory endothelial phenotype and genes regulating cell motility and angiogenic processes. Reactome database analysis identified 91 pathways that were active in endothelial cells at D28 compared to D0 (Table S13). Pathways of long-term induced genes relate to fibrosis, inflammation, hemostasis and thrombosis, proliferation and circadian clock (Fig. 4c, d).

**Table 3:** Day 28 post-MI gene expression changes and the corresponding biological processes and cellular functions.

Day 28 post-MI upregulated genes and biological processes in endothelial cells					
ECM/Fibrosis	Inflammation	Metabolism	Angiogenesis/Motility	Proliferation/Growth	Hemostasis/Thrombosis
*ECM proteins*	*Chemokines/Cytokines*	*Nucleotide Biosynthesis*	*Angiogenesis regulators*	*Cell Cycle/Cell growth*	*Coagulation/Thrombosis*
Col3a1**, Col15a1***, Fbln1, Lamc2, Mfap4, Mfap5***, Postn, Thbs1, Thbs4, Tnc, Vcan	Cxcl1***, Cxcl2, Cxcl10, Il6*, Lif	Mthfd2	Bmpr1a, Lrg1**, Dkk3, Esm1***, Inhbb, Pth1r***, Runx1	Ccno***, Cdkl2***, Fgf1***, Myc	Pdpn, Sele**, Selp**, Serpine1*** (PAI-1), Serpine2***, Thbs1
*ECM processing/collagen-crosslinking enzymes*	*Receptors/Signaling*	*Amino acid biosynthesis*	*Migration/Tube formation*	*RNA synthesis*	
Adam12, Ctsk***, Loxl1, Loxl3, Mmp3, Timp1	Ccr7***, Csf2rb, Csf2rb2, H2-Aa***, Lrg1**	Aldh18a1	Itga2, Itgbl1, Sema3c, Slit2, Tubb6	Polr2h, Polr2k***	
*Collagen fibril assembly*	*Cell adhesion & recruitment*	*Amino acid transporters*			
Fmod***, Lum***	Plvap**, Sele**, Selp**, Vcam1**	Slc1a4			

				**Hypoxia/Stress**	**Circadian Clock**
*Profibrotic factors*	*Prostaglandin biosynthesis*	*Membrane transporters*		*Oxidative Stress*	*Circadian Pathway*
Ltbp2, Sfrp1, Sfrp2	Ptgis***, Ptgs2 (Cox-2)	**Slc10a6**		Akr1b8, Aldh1a1, Aldh1a2, Aox1***	Bhlhe40***, Bhlhe41***, Dbp***, Hlf***, Hif1α, Nr1d1***, Nr1d2***, Per2, Per3***, Tef***
		*Protein modification enzymes*		*Hypoxia*	
		Hs6st2***		Hif1α	
		*Calcium/phosphate homeostasis*		*Stress/Apoptosis*	
		Stc1***		Acer2***, Bnip3, Dnajb9***	

Day 28 post-MI downregulated genes and biological processes in endothelial cells					
Vascular tone	Vascular homeostasis	Proliferation/Growth	Metabolism		
*Filament proteins/Contraction*	*GPCR receptors*	*Proliferation*	*Membrane transporters*		
Myh6*, Myl4*, Mylk3	Adra2c***, Cnr2*, **F2rl1**, Gper1, Tas1r3***	Sox12***, Wnt9b***, Tymp***	Aqp1, Slc16a11, Slc38a3		

**ECM/Fibrosis**					
*ECM proteins*	*Nuclear hormone receptors*		*ABC transporters*		
Fbln2***	Thra*		Abcb9		

**Hypoxia/Stress**	**Inflammation**	**Signaling Regulation**	**Circadian Clock**		
*Stress/Apoptosis*	*Transcriptional Regulation*	*Negative Regulators*	*Circadian pathway*		
Alkbh2***, Arntl (Bmal1)	Foxo4***	Asxl3*, Ltbp4*, Bost	Arntl (Bmal1), Cry1		
	*Cell Transmigration*				
	Cldn5***				

*Marks genes whose post-MI expression patterns are depicted in Fig. 2.

**Marks genes whose post-MI expression patterns are depicted in Fig. 3.

***Marks genes whose post-MI expression patterns are depicted in Fig. 4.

Genes in Bold are primarily induced or suppressed at D28.

All genes in D28 Table represent genes whose post-MI expression levels did not return to baseline.

The list of the top 20 downregulated genes at D28 is shown in Fig. 4e and the complete list in Table S14. The downregulation of genes involved in contractility and vascular tone observed at D2 and D7 extended to D28 (Fig. 4f; Table S15). Similarly, expression of several hormone receptors remained lower than baseline values, suggesting that EC homeostatic functions were not fully restored. There was also downregulation of genes involved in cell proliferation and inflammation (Fig. 4g).

The most striking characteristic of late post-MI EC expression changes was the deregulation of 12 genes in the circadian pathway, which is critical for rhythmic control of cardiovascular functions^36^. Induction or suppression of circadian clock genes in ECs started at D2 after MI, but gradually amplified and reached peak values at D28 (Fig. 4d; Table 3).

In brief, gene expression profiling data at D28 demonstrated that the MI injury left long-term marks on ECs linked to acquisition of proinflammatory, prothrombotic and profibrotic properties, loss of circadian rhythmicity, and incomplete restoration of endothelial quiescence as manifested by the aberrant expression of genes involved in metabolism and cell proliferation.

### Sh2d5 promotes endothelial cell growth and motility

**Figure 5:**
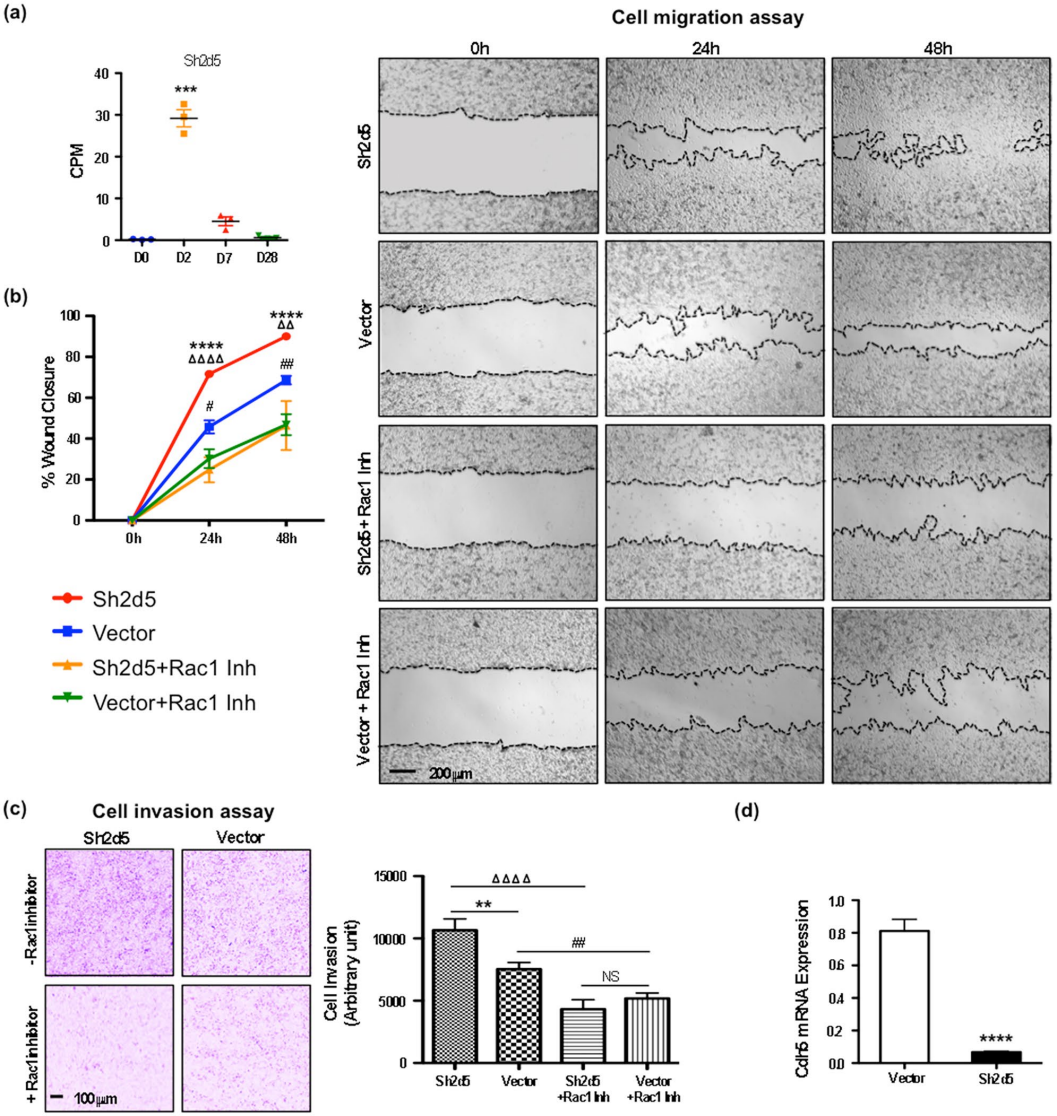
Sh2d5 is induced after MI and positively regulates endothelial cell growth and migration. (**a**) Sh2d5 is the highest-induced gene in endothelial cells at D2 after MI. Statistical analysis of the Sh2d5 expression in RNAseq data was performed with Partek’s Gene Specific Analysis (GSA) multimodal estimation, n = 3 biological replicates. *** *p* < 0.001 comparing D2 expression levels to D0 values at baseline without MI. (**b**) Overexpression of Sh2d5 in cultured endothelial cells promotes growth and migration in a wound closure/scratch assay. The Sh2d5 effect is abolished by addition of the Rac1 GTPase chemical inhibitor Z62954982 in the culture media. Representative images of the scratch assay at 24 h and 48 h are depicted in the left panels, n = 6 biological replicates. 24 h: **** *p* < 0.0001, Shd25 vs vector; ^ΔΔΔΔ^ *p* < 0.0001, Sh2d5 vs Sh2d5 + Rac1 Inh; ^#^ *p* < 0.05, vector vs vector + Rac1 Inh. 48 h: **** *p* < 0.0001, Shd25 vs vector; ^ΔΔ^ *p* < 0.01, Sh2d5 vs Sh2d5 + Rac1 Inh; ^##^ *p* < 0.01, vector vs vector + Rac1 Inh. (**c**) Overexpression of Sh2d5 in cultured endothelial cells promotes migration in a transwell cell invasion assay, n = 9 biological replicates. The Sh2d5 effect is abolished by addition of the Rac1 GTPase chemical inhibitor Z62954982. ** *p* < 0.001, Shd25 vs vector; ^ΔΔΔΔ^ *p* < 0.0001, Sh2d5 vs Sh2d5 + Rac1 Inh; ^##^ *p* < 0.01, vector vs vector + Rac1 Inh. (**d**) RT-qPCR analysis shows that overexpression of Sh2d5 in cultured endothelial cells suppresses the expression of the endothelial-specific cell adhesion gene *VE-cadherin* (*Chd5*), n = 9 (3 biological and 3 technical replicates). **** *p* < 0.0001, Shd25 vs vector. NS: not significant. Statistical analyses for cell migration assay (**a**), cell invasion assay (**c**) and qPCR (**d**) were performed with unpaired *t*-test for comparison between two groups using GraphPad Prism software.

The highest induced gene in ECs by165-fold at D2 was the mammalian-specific *Sh2d5* gene (Fig. 5a; Table S4). Induction levels decreased at later stages, but remained higher than baseline throughout the repair process. Sh2d5 binds to and blocks the Breakpoint cluster region (Bcr) protein^37^. Bcr is a GTPase-activating protein (GAP) for Rac1 GTPase of the Rho family, promoting conversion of GTP to GDP, thereby inactivating Rac1.

Because of the prominent induction of Rho pathway components post-MI, including Bcr (Table 2), and the capacity of Sh2d5 to inhibit Bcr, which blocks Rac1 activation, we tested whether Sh2d5 promotes Rac1-regulated endothelial motility using two approaches, a wound closure (or scratch) and a transwell cell invasion assay. Human ECs in culture were transfected with a plasmid expressing the human *Sh2d5* gene. Empty vector plasmid transfected cells served as a control (Figs. S2, S3). In the scratch assay, which measures a combination of proliferation and motility, we found that *Sh2d5* overexpression accelerated wound closure (Fig. 5b). The transwell cell invasion assay, which directly assesses EC migration and invasiveness, showed that *Sh2d5* overexpression promoted matrix invasion (Fig. 5c). These positive effects were abolished when the specific Rac1 chemical inhibitor Z62954982 was added after transfection, indicating that Rac1 activation is required for the Sh2d5 effects (Fig. 5b, c). Further supporting its role in enhancing EC motility, *Sh2d5* overexpression suppressed the major endothelial-endothelial cell adhesion molecule *VE-Cadherin* (*Cdh5*; Fig. 5d). Together, our data indicate that Sh2d5 is a novel regulator of the Rac-1 signaling axis in ECs.

## Discussion

ECs are instrumental for the repair of cardiac tissue following MI, providing a pertinent entry point to improve cardiac function after acute ischemic injury. To further our understanding of the EC response after MI, we have undertaken a comprehensive analysis of the endothelial transcriptome at landmark time points after MI. Our key findings are: (1) ECs acquired stage-specific phenotypes after MI, reflecting temporal roles in cardiac wound healing processes; (2) MI had long-term sequelae on ECs; (3) MI disrupted endothelial homeostatic functions, vascular tone, and circadian rhythmicity, and these deficits did not fully resolve with time; (4) ECs upregulated a broad repertoire of genes encoding ECM proteins and ECM remodeling enzymes during scar tissue formation; and, (5) *Sh2d5* was the highest induced gene in ECs after MI and promoted growth and migration through activation of the Rac1 GTPase pathway, which regulates cell–cell adhesion, cell motility, proliferation and glucose uptake.

Our approach assessed RNA level changes after MI in 12,503 expressed genes, which represent the majority (~ 90%) of cardiac EC transcripts. We identified 1,699 DE genes with distinct patterns of induction or suppression and assigned them to ten pathological and physiological processes. The number of gene expression changes related to metabolism, cell growth, stress and vascular tone peaked early and declined thereafter, whereas, deregulation of circadian clock genes increased at late stages. Variation in genes associated with angiogenesis, as well as profibrotic and prothrombotic phenotypes, peaked during scar formation, whereas deregulation of proinflammaory genes and genes involved in vascular homeostatic functions took place throughout the examined time points (Fig. S4). Most gene expression changes, in 1232 of the 1699 DE genes (72.5%), were temporal and specific to the corresponding repair stage.

Recent single cell RNAseq (scRNAseq) studies have underscored the diversity and heterogeneity of endothelial subpopulations at different stages after MI^38,39^. Furthermore, immunostaining has been extensively used to identify regional specificity in the expression of genes induced after MI. For example, we and others have shown that induction of mesenchymal markers takes place primarily in ECs in the peri-infarct border zone and within the area of the scar^7,39,40^. Similarly, analysis of cell cycle markers at different stages after MI showed EC proliferation is confined in the peri-infarct border zone and peaks around D3^39^. Here, we show that this regional specificity in endothelial response to MI extends to proinflammatory markers that were induced at higher levels in proximal- versus distal-to-infarct areas.

Our study further corroborated findings from scRNAseq data and conventional RNA expression profiling regarding the timing of post-MI induction of individual genes, for example *Sele*, *Angpt2*, *Plvap*, *Il-6*, or *Snail*, to name a few. It also confirmed and expanded findings regarding the sequential induction of genes involved in the S and M phases of the cell cycle, the post-MI metabolic adaptation of ECs, and the upregulation of angiogenic and mesenchymal markers during neovascularization and fibrosis^38,39^.

We discovered that the endothelial transcriptome did not fully return to pre-injury levels after the repair has been completed. Instead, we observed long-term acquisition of prothrombotic, proinflammatory and profibrotic properties, which may increase the risk and incidence of subsequent ischemic events, chronic heart inflammation, and vascular dysfunction. In addition, the endothelial circadian rhythm appears to be disrupted, as indicated by the deregulated expression of numerous components of the circadian clock. Peripheral circadian clocks in cardiomyocytes, ECs, smooth muscle cells and stromal fibroblasts regulate cardiovascular functions, such as cardiac contractility, heart rate, vascular tone, blood pressure, as well as proinflammatory and prothrombotic endothelial phenotypes^36^. However, the specific mechanisms of the endothelial circadian clock in post-MI repair processes are currently unknown and require future investigations.

Our data also revealed that MI disrupted vascular tone and vascular homeostasis with suppression of genes that control Ca^2+^ dynamics and contractility and decreased expression of membrane and nuclear receptors that regulate endothelial responses to various hormones. Although suppression of relevant genes was in some instances transient, a subset of downregulated genes did not return to pre-MI levels, suggesting both short- and long-term disruptions of normal vascular functions. These abnormalities may eventually lead to sustained endothelial dysfunction, a key contributor to post-MI HF^41^.

We found that the highest induced gene after MI encoded the Sh2d5 protein, the function of which has not been previously investigated in ECs. Sh2d5 is the latest evolutionary addition to the family of Src homology 2 (SH2) domain containing proteins that mediate protein–protein interactions among tyrosine-phosphorylated substrates^42^. Initial studies suggested that Sh2d5 adds an extra layer of positive regulation in Rac1 GTPase and Stat3 signaling pathways^37,43^. Our functional analyses in cultured ECs indicated that Sh2d5 is a novel regulator of signaling pathways involved in EC growth and motility, providing a promising new target to modulate EC function and improve cardiac tissue repair after MI.

In summary, our study revealed a pronounced compartmentalization in the EC response to MI, as manifested in the synchronization of cell cycle phases, stage-specific changes in metabolic pathways, angiogenesis, and acquisition of mesenchymal characteristics, as well as long-lasting effects of the initial ischemic injury. These findings underscore the necessity for precisely-timed interventions in order to optimize cardiac tissue repair and improve ischemic disease outcomes. Comparison of mechanisms and biological processes that are activated or suppressed in animal models and human patients after MI have revealed that animal models recapitulate both the timing and location of the ischemic injury effects in the human heart^44^. Therefore, our study will potentially provide both new targets and endothelial biomarkers that that could be utilized to improve clinical MI outcomes.

## Materials and methods

### Coronary artery ligation and echocardiography

All animal experiments were approved by the Vanderbilt University Institutional Animal Care & Use Committee (IACUC Protocol# M1600041-02) and performed in accordance to the NIH Guide for the Care and Use of Laboratory Animals and ARRIVE guidelines. Experimental MI in mice was performed as we previously described^6,7^. Briefly, 4–6 months-old C57BL/6 male mice (JAX) were subjected to open chest surgery, a 10-0 nylon suture was placed through the myocardium into the anterolateral left ventricular wall around the left anterior descending (LAD) artery, and the vessel was permanently ligated. During surgery, mice were kept under anesthesia with continuous inhalation of 1–2% isoflurane. After surgery, mice were treated with analgesics by subcutaneous injection of 5–10 mg/kg ketoprofen every 24 h. Mice were euthanized at different post-MI time points with isoflurane overdose and the left ventricle (LV) was dissected for further analyses.

Echocardiography was performed in conscious mice using the Vevo2100 ultrasound with the MS-400 transducer (VisualSonics, Canada). The LV was located in B-Mode and traced over five consecutive beats in M-Mode. LV internal dimension and volume in diastole and systole (LVIDd, LVIDs, LVvold, LVvols) were calculated in M-Mode from the short axis and used to derive fractional shortening (FS) and ejection fraction (EF) values^6–8^.

### Isolation of cardiac ECs by Flow Activated Cell Sorting (FACS)

To prepare single cell suspensions of ECs for RNA sequencing, whole LV tissue was dissociated into small pieces followed by digestion with Collagenase II (Worthington, USA; Cat. No. LS004177) and DNase I (AppliChem, Germany; Cat. No. APA3778.0500) and then incubated at 37 °C for 30 min. Collagenase II working solution was freshly prepared by dissolving Collagenase II to 600 U/ml and DNase I to 60 U/ml in Hanks' Balanced Salt Solution (HBSS). The digested cell suspension was applied over pre-separation filters (30 μm) (Miltenyi Biotec, Germany; Cat. No. 130-041-407) to remove cardiomyocytes and cell clumps, and the flow-through was centrifuged at 300 × g for 10 min. The cell pellet was resuspended in 1 ml PEB buffer containing phosphate buffer saline (PBS, pH 7.2), plus 2 mM EDTA and 0.5% BSA and 10 ml of 1× Red Blood Cell (RBC) lysis buffer (Miltenyi Biotec; Cat. No. 130-094-183). Cells were incubated for 2 min to remove RBC. Cells were pelleted by centrifugation at 300 × g for 10 min, resuspended in 2 ml 1× PEB buffer and counted on a hemocytometer. CD45 microbeads (Miltenyi Biotec; Cat. No. 130-052-301; 10 μl microbeads/10^7^ cells) were added to the cells and incubated for 15 min. The MS column was placed in the magnetic field of the MiniMACS separator using the MACS Multistand. Cells were passed through the magnetic column in order to remove CD45^+^ immune cells. The remaining CD45^-^ effluent cells, consisting primarily of ECs, fibroblasts, and pericytes, were collected by centrifugation, resuspended in PBS and TruStain FcX™ (anti-mouse CD16/32 antibody from BioLegend, U.S.A.; Cat. No. 101320) was added to block non-specific binding. Cells were then stained with PE-conjugated anti-mouse CD31 (BioLegend; Cat. No. 102408) and Alexa Fluor 700-conjugated anti-mouse Ly-6A/E (Sca-1) (BioLegend; Cat. No. 108142) antibodies. After incubating in the dark for 20 min, 7-AAD Viability Staining Solution (Biolegend; Cat. No. 420404) was added and CD31/Sca1 double positive ECs were separated as a pure population by FACS and lysed for RNA preparation using the RNeasy Mini kit (Qiagen, Germany; Cat. No. 74106).

### Bulk-RNAseq and bioinformatics analysis

RNA samples were submitted to the Vanderbilt Technologies for Advanced Genomics (VANTAGE) core for quality controls (QC), including sufficient RNA concentration, 260/280 ratio of 2, and high RNA Integrity Number (RIN) as confirmed with an Agilent Bioanalyzer (Agilent Technologies Inc., USA). Poly(A) RNA Sequencing was performed at the VANTAGE core using Illumina NovaSeq6000 (Illumina Inc., USA) on paired-end-150 flow cell runs at ~ 40 million PF reads per sample. Raw reads (fastq files) were uploaded to the Partek Flow server and pre-alignment quality assessment was performed (Partek Inc., USA). Sequences were aligned to the mm10 assembly of the mouse genome using STAR 2.5.3a and resulting reads quantified at the gene level to Ensembl transcripts 93 using Partek’s expectation–maximization (E/M) annotation model. Gene counts were normalized to the total read count per sample and then log-transformed (with an offset of 0.0001).

Hierarchical clustering and PCA were performed on normalized and log-transformed counts using Partek Genomics Suite 7.19.1125. Venn diagrams were used to establish the number of stage-specific and shared genes in each post-MI time point using the online tool Venny (https://bioinfogp.cnb.csic.es/tools/venny/). Volcano plots were created to visualize differentially expressed genes at different post-MI time points using the online tool VolcaNoseR (https://huygens.science.uva.nl/VolcaNoseR/). Volcano plot analysis for each post-MI time-point was performed by comparing to D0, with fold change threshold of ≥ 1.5 and *p* ≤ 0.05. Enrichment pathway analysis was done using the Reactome Pathways tool in the online Enrichr database (https://maayanlab.cloud/Enrichr/#).

### Cell culture, transfection and Rac1 inhibitor treatment

Dermal Human Microvascular Endothelial cells (HMEC-1), which had been previously immortalized by transfection of the simian virus 40 large T antigen gene, were cultured as we have previously described^7,8,45^ in Media 199 (Gibco, U.S.A.; Cat. No. 11150-059) containing 15% FBS (Gibco; Cat. No. 26140-079), 10 U/ml Heparin (Sigma, U.S.A.; Cat. No. H3149), and 30 μg/ml Endothelial cell growth supplement (Sigma; Cat. No. E2759). Trypsinization of the cells was performed using TrypLE Express (Gibco; Cat. No. 12604-013).

Cells were transfected using Lipofectamine LTX with Plus Reagent (ThermoFisher Scientific, U.S.A.; Cat. No. 15338100) in Opti-MEM reduced Serum Medium (ThermoFisher Scientific; Cat. No. 31-985-070), following the manufacturer’s protocol. Cells were transfected with either Sh2d5-expressing plasmid [Human Sh2d5 (NM_001103161) C-end GFP-Tagged ORF Clone; Cat No. RG211583] or pCMV6-AC-GFP Mammalian Expression Vector (Cat. No. PS100010) from OriGene, USA. Transfection efficiency was estimated using ImageJ for quantification of fluorescent (i.e., transfected) cells as a fraction of the total cell number using cell counts in 2 areas from 3 independent transfections (n = 6) (Fig. S2). Successful expression of *Sh2d5* RNA and Sh2d5 protein were verified by qPCR and Western blotting, respectively (Figs. S2, S3). Following transfection, cells were either kept untreated or treated with Insolution Rac1 inhibitor II, Z62954982 (Calbiochem, U.S.A.; Cat. No. 553512) to a final concentration of 40 μM.

### Wound closure assay

Wound closure (or scratch) assay was performed with HMEC-1 cells. Growing cells were plated at 60–70% confluency in 6-well plates before the day of transfection. Next day, cells were transfected with either Sh2d5 or control plasmid using Lipofectamine Plus reagent. When transfected cells reached 95% confluency, the scratch was introduced into the confluent monolayer of the cells using a p200 pipette tip. After the scratch, the cell monolayer was gently washed using 1× PBS to remove detached cells. Then, fresh medium with or without Rac1 inhibitor was added. The 6-well plates were incubated at 37 °C in a CO_2_ incubator for 24 and 48 h and images of wound closure were acquired under a phase-contrast microscope. The area of wound closure was measured using ImageJ software.

### Cell invasion assay

Cell invasion assay was performed with HMEC-1 cells. Corning Matrigel Basement Membrane Mix (MilliporeSigma, U.S.A.; Cat. No. CL356234) was diluted to 1:1 concentration using serum free growth medium. Transwell chambers were prepared by inserting permeable cell culture inserts in 6-well plates. Permeable inserts with 3 μm pore size were purchased from Celltreat, USA (Cat. No. 230603). Into the upper transwell chamber, 400 μl diluted Matrigel was added and incubated overnight at 37 °C in order to gel. Media with serum was added to the lower chamber and serum-free media was added to the upper chamber. Cells were added to the upper chamber and allowed to invade across the Matrigel for 48 h. To assess cell invasion, media was removed and transwell plate chambers were washed twice using 1× PBS. Cells were fixed using 3.7% formaldehyde in PBS at room temperature (RT) for 2 min. The formaldehyde was removed and the transwell chamber was washed twice with 1× PBS. Cells were permeabilized with 100% methanol at RT for 20 min. Methanol was removed, and the chamber was washed twice with 1× PBS. Cells were then stained with 0.25% crystal violet (Sigma, USA; Cat. No. C0775) in the dark at RT for 15 min. Staining solution was removed, and the insert was washed twice with 1× PBS. Non-invading cells were scraped off the upper part of the insert using a cotton swab. EC invasion was visualized under a microscope followed by quantification using ImageJ software.

### Western blotting

Western blotting was performed using whole cell protein extract from HMEC-1 cells. In brief, cells were harvested and washed with 1× PBS. Cell pellets were resuspended in RIPA lysis buffer (Sigma Cat. No. R0278) containing protease inhibitor cocktail (Roche Cat. No. 4693116001) for 5 min on ice followed by centrifugation at 13,000 × g for 15 min at 4 °C. The supernatant was used as whole cell lysate.

Protein concentration in cell lysates was determined using the Pierce BCA protein assay kit (ThermoFisher Scientific, Cat. No. 23225), according to the manufacturer’s protocol. Equal amounts of protein per sample were loaded and separated on SDS-PAGE gels and transferred to nitrocellulose membrane (Cytiva Amersham Protran Premium 0.2 μm NC, Cat. No. A30057702). After blocking the membrane with Intercept TBS blocking buffer (LI-COR, Cat. No. 927-60001) the membrane was hybridized with the respective primary and secondary antibodies. PageRuler Plus Prestained Protein Ladder (ThermoFisher Scientific, Cat. No. 26619) was used as protein molecular weight marker. The protein bands were visualized, and images were captured using Odyssey Imaging System. The following antibodies were used for Western blotting: Sh2d5 antibody (Invitrogen Cat. No. PA5-101883); Gapdh antibody (Invitrogen Gapdh Loading Control Monoclonal Antibody (GA1R) (Cat. No. MA5-15738); Alexa Fluor® 488 AffiniPure Donkey Anti-Mouse IgG (H + L), Jackson Immunoresearch, Cat. No. 715-545-150; and Alexa Fluor® 790 AffiniPure Donkey Anti-Rabbit IgG (H+L), Jackson Immunoresearch, Cat. No. 711-655-152. Antibodies were used in 1:1000 dilution.

### RNA analysis by Reverse Transcription quantitative PCR (RT-qPCR)

RNA was extracted from HMEC-1 cells using the Qiagen RNeasy Mini kit. Reverse transcription was performed using the ThermoFisher Scientific RevertAid RT Kit (Cat. No. K1691) according to the manufacturer’s protocol. qPCR was performed using PowerUp SYBR Green Master Mix (ThermoFisher Scientific; Cat. No A25742) in a C1000 Thermal Cycler (BioRad, USA). Gapdh primers were used as internal control and relative gene expression levels were determined using the 2^−ΔΔCt^ method^7,8^. Experiments were done in triplicates. Primers used for human *Cdh5* gene were forward primer 5′-TCTCCCCTTCTCTGCCTCACCTG-3′ and reverse primer 5′-CCCTCTCTGTTGACTGATGCCACTTC-3′; primers used for human *Gapdh* gene were forward primer 5′-CAAGGTCATCCATGACAACTTTGGT-3′ and reverse primer 5′-CAGTAGAGGCAGGGATGATGTTCTG-3′; primers for human *Sh2d5* gene were forward primer 5′-GGGAGGGTGAGGTGCTGCTGAT-3′ and reverse primer 5′-TGGCTGGCTGCCCACAAAGAGG-3′.

### Statistical analysis

Statistical analysis of RNAseq data was performed with Partek’s Gene Specific Analysis (GSA) multimodal estimation to identify differentially expressed genes with cutoff values of *p* ≤ 0.05 and fold change ≥ 1.5. Statistical analyses for transfection efficiency, qPCR, wound closure assays and cell invasion assays were performed with unpaired *t*-test for comparison between two groups using GraphPad Prism software and data are represented as the mean ± SEM. *p* ≤ 0.05, values were considered significant.

### Supplementary Information

Supplementary Figures.

Supplementary Table S1.

Supplementary Table S2.

Supplementary Table S3.

Supplementary Table S4.

Supplementary Table S5.

Supplementary Table S6.

Supplementary Table S7.

Supplementary Table S8.

Supplementary Table S9.

Supplementary Table S10.

Supplementary Table S11.

Supplementary Table S12.

Supplementary Table S13.

Supplementary Table S14.

Supplementary Table S15.

## Data Availability

The primary RNA sequencing datasets generated during the current study are available in the National Center for Biotechnology Information (NCBI) Sequence Read Archive (SRA) under Accession Numbers SAMN39923325, SAMN39923326, SAMN39923327, SAMN39923328, SAMN39923329, SAMN39923330, SAMN39923331, SAMN39923332, SAMN39923333, SAMN39923334, SAMN39923335, and SAMN39923336. All data analyzed during this study are included in this published article (and its Supplementary Information files) and will be freely available for non-commercial purposes.
