# Supplementary material for: Transcriptome analysis of cardiac endothelial cells after myocardial infarction reveals temporal changes and long-term deficits
Source: Sci Rep. 2024 May 1;14:9991. doi: 10.1038/s41598-024-59155-8 (PMC11063162; doi:10.1038/s41598-024-59155-8)
Supplement: Supplementary file 1 — Supplementary Figures. [file 41598_2024_59155_MOESM1_ESM.pdf]

Fig. S1. Spatial regulation of-pro-inflammatory endothelial marker Sele induction after MI

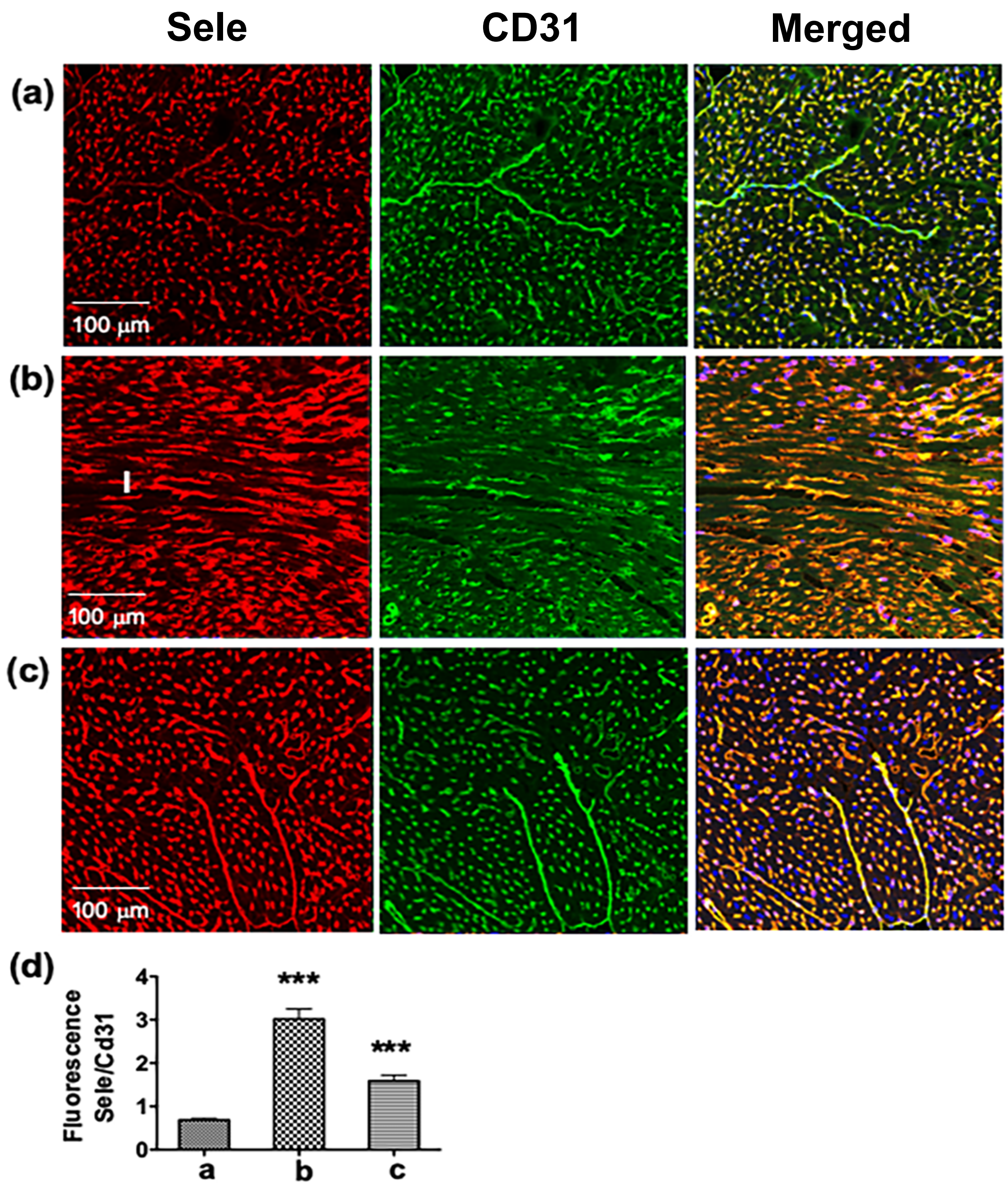

Histological analysis of Sele (red) and CD31 (green) proteins in cardiac tissue sections. Cell nuclei were stained with DAPI (blue in Merged).

(a) At D0 normal mouse hearts, Sele was universally expressed in endothelial cells, similarly to CD31, but at relatively lower levels compared to CD31. (b,c) Sele protein increased at D2 post-MI, relatively to CD31. Increased Sele expression was detected on all endothelial cells, but induction levels were higher in and around the infarct site [I; (b)] compared to distal areas (c). (d) Quantification of Sele fluorescence intensity relative to CD31 using image J software. Sele to CD31 ratio at D0 was 0.7. Sele levels showed approximately 5-fold increase around the infarct site, versus 2-fold increase in distal areas, relatively to CD31. ***$p<0.001$ compared to D0. Size bars represent 100 μm.

Fig. S2. Transfection efficiency of the HMEC-1 endothelial cell line and Sh2d5 overexpression

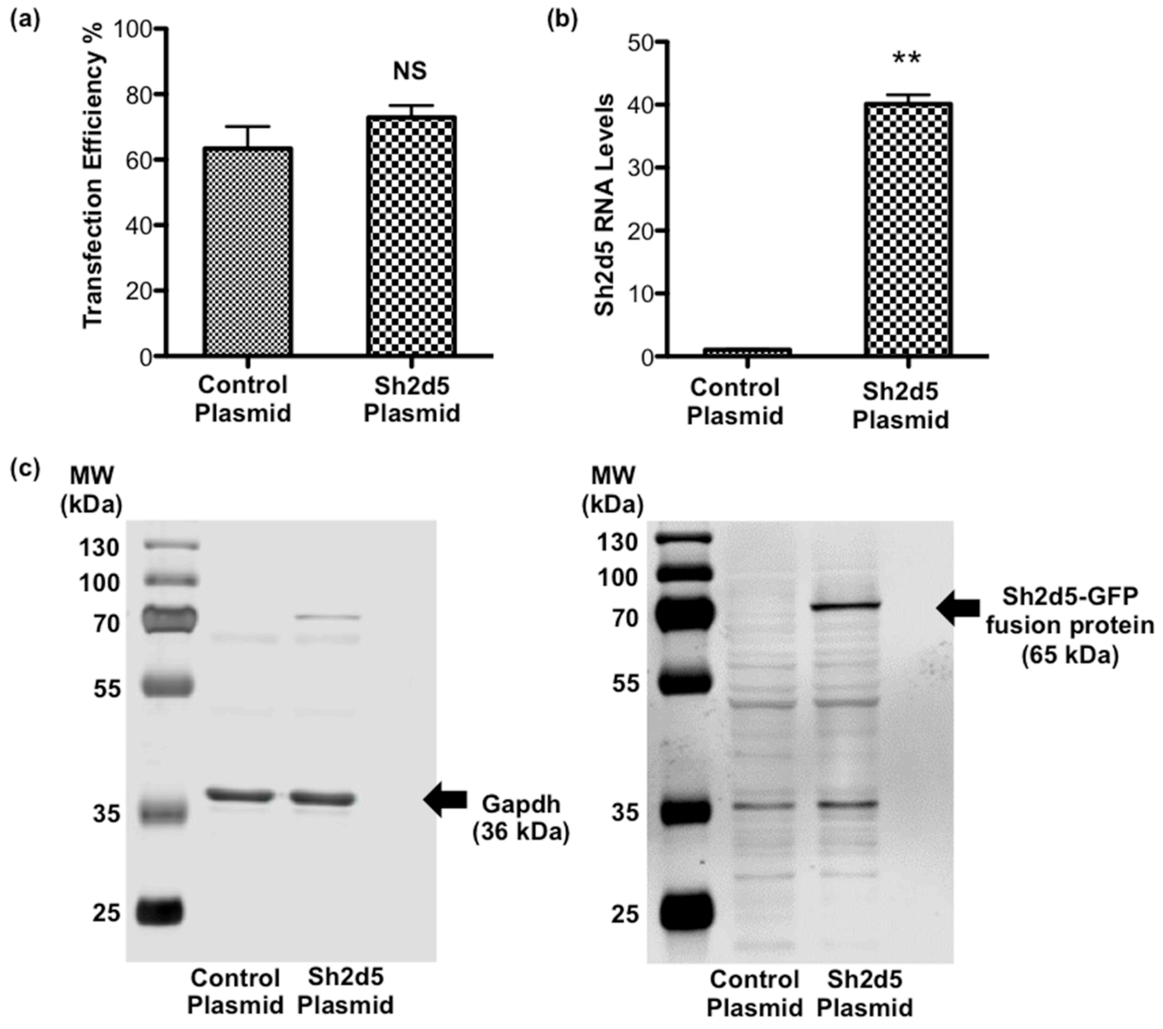

(**a**) The HMEC-1 cell line was transfected with Sh2d5-expressing plasmid and empty vector control. Both plasmids carry the GFP gene, allowing quantification of transfection efficiency as percentage of GFP-positive to total cell numbers. (**b**) qPCR analysis shows robust increase in Sh2d5 RNA expression levels in Sh2d5-plasmid transfected HMEC-1 cells compared to vector control. (**c**) Western blotting reveals strong increase of Sh2d5 protein levels in Sh2d5-plasmid transfected HMEC-1 cells compared to vector control. Protein lysates of HMEC-1 transfected cells were analyzed with antibodies recognizing Sh2d5 protein and Gapdh as loading control. The Sh2d5 C-tagged GFP fusion protein band of approximately 65 kDa is evident only in Sh2d5-expressing plasmid transfected cells. Original gel blots are presented in Supplementary Fig. S3.

Fig. S3. Original gel blot images

Sh2d5 blot image

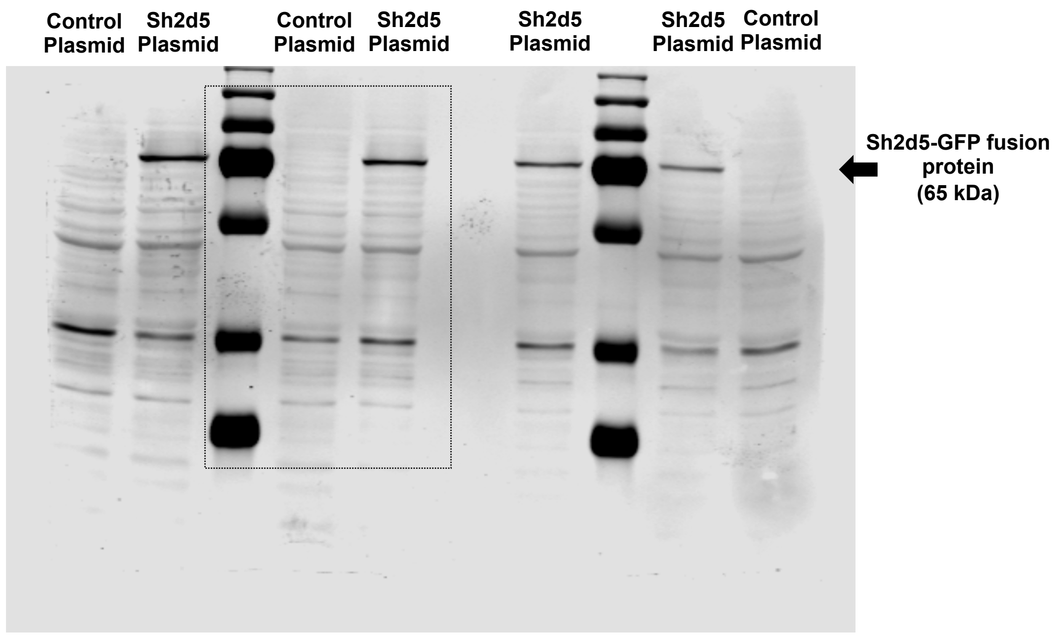

Gapdh blot image

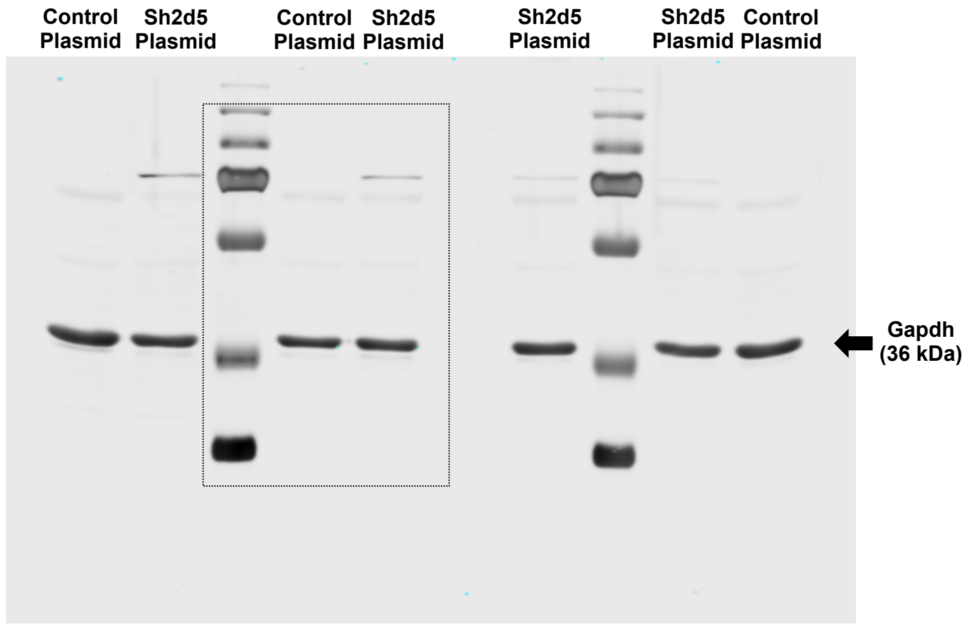

Original images of gel blots shown in Fig. S2. Dotted lines indicate the cropped parts used in Fig. S2.

Fig. S4. Dynamics of phenotypic changes in cardiac endothelial cells during the MI repair process

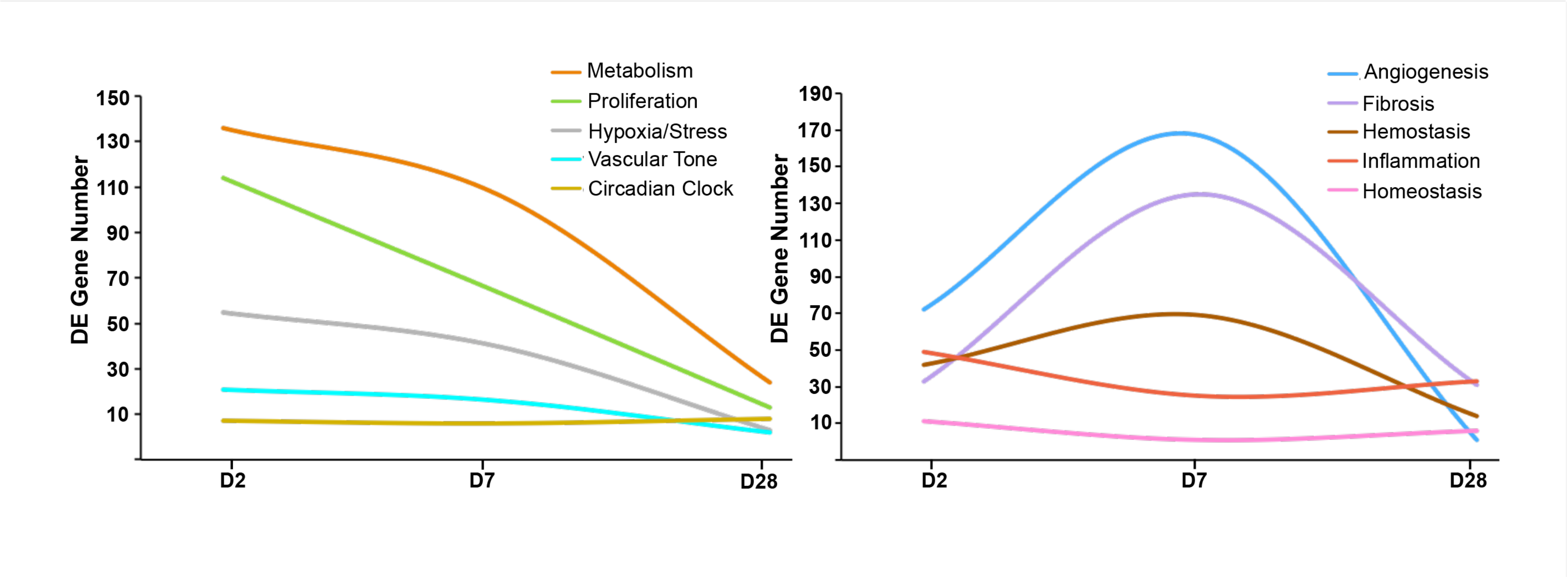

Plots of gene numbers associated with specific biological processes (marked by color lines) at distinct time points after MI.
